# Atmospheric CO_2_ forcing on Mediterranean biomes during the past 500 kyrs

**DOI:** 10.1038/s41467-023-37388-x

**Published:** 2023-03-25

**Authors:** Andreas Koutsodendris, Vasilis Dakos, William J. Fletcher, Maria Knipping, Ulrich Kotthoff, Alice M. Milner, Ulrich C. Müller, Stefanie Kaboth-Bahr, Oliver A. Kern, Laurin Kolb, Polina Vakhrameeva, Sabine Wulf, Kimon Christanis, Gerhard Schmiedl, Jörg Pross

**Affiliations:** 1 Institute of Earth Sciences, Heidelberg University, Heidelberg, Germany; 2 Institute des Sciences de l’Évolution, Université de Montpellier, CNRS, IRD, EPHE, Montpellier, France; 3 Institut d’Écologie et des Sciences de l’Environnement de Paris (iEES Paris), Sorbonne Université, Paris, France; 4 School of Environment, Education and Development, The University of Manchester, Manchester, UK; 5 Department of Molecular Botany, Institute of Biology, University of Hohenheim, Stuttgart, Germany; 6 Center for Earth System Research and Sustainability, Institute of Geology, Hamburg University, Hamburg, Germany; 7 Leibniz Institute for the Analysis of Biodiversity Change (LIB), Hamburg, Germany; 8 Department of Geography, Royal Holloway University of London, London, UK; 9 Parlamentsstraße 32, 60385 Frankfurt am Main, Germany; 10 Senckenberg Biodiversity and Climate Research Centre (SBiK-F), Senckenberg Gesellschaft für Naturforschung, Frankfurt am Main, Germany; 11 Institute of Geosciences, University of Potsdam, Potsdam-Golm, Germany; 12 School of the Environment, Geography and Geosciences, University of Portsmouth, Portsmouth, UK; 13 Department of Geology, University of Patras, Rio, Greece

**Keywords:** Palaeoclimate, Palaeoecology

## Abstract

There is growing concern on the survival of Mediterranean forests under the projected near-future droughts as a result of anthropogenic climate change. Here we determine the resilience of Mediterranean forests across the entire range of climatic boundary conditions realized during the past 500 kyrs based on continuous pollen and geochemical records of (sub)centennial-scale resolution from drillcores from Tenaghi Philippon, Greece. Using convergent cross-mapping we provide empirical confirmation that global atmospheric carbon dioxide (CO_2_) may affect Mediterranean vegetation through forcing on moisture availability. Our analysis documents two stable vegetation regimes across the wide range of CO_2_ and moisture levels realized during the past four glacial-interglacial cycles, with abrupt shifts from forest to steppe biomes occurring when a threshold in precipitation is crossed. Our approach highlights that a CO_2_-driven moisture decrease in the near future may bear an impending risk for abrupt vegetation regime shifts prompting forest loss in the Mediterranean region.

## Abstract (web summary)

A 500 kyr long record of vegetation change from SE Europe demonstrates that forest resilience is lost when precipitation decreases below a threshold limit, and highlights the vulnerability of Mediterranean forests to near-future climate change

## Introduction

The Mediterranean region is characterized by a mosaic of subtropical and temperate forests thriving under water-stressed conditions^1,2^. Besides representing vulnerable biodiversity hotspots^3^, these Mediterranean forests provide a wide range of critical ecosystem services such as support of soil, regulation of regional climatic and hydrological conditions, and supply of food and timber^4^. In light of yet unabated anthropogenic CO_2_ release, there is increasing concern on the resilience of these forest biomes to the recurring droughts^5,6^ as they are projected for the Mediterranean region in the next 70 years^4,7^. Climate scenarios predict structural and compositional changes in Mediterranean forest biomes unprecedented in the Holocene that will cause disruption of ecosystem services and trigger biodiversity loss^8,9^. To assess the resilience of Mediterranean forest biomes to future climate forcing, it is essential to determine the nature and rate of change, and forcing mechanisms that these biomes have experienced under the natural range of climate conditions during the Quaternary^10^.

Here, we analyze a unique, exceptionally long record of Mediterranean forest dynamics based on centennial-scale-resolution (~200 years) pollen data from drillcores from Tenaghi Philippon (Greece; Supplementary Fig. 1) covering the past 500 kyrs continuously^11^. Augmented by decadal-scale-resolution geochemical data (~30 years) that record precipitation variability, our datasets resolve vegetation and moisture dynamics at timescales that do justice to the tempo of anthropogenic climate change. The Tenaghi Philippon cores have excellent chronostratigraphical control based on the integration of radiocarbon dating, tephrochronology and orbital tuning (Methods), and cover the full range of climatic boundary conditions realized during the past four glacial/interglacial (G/I) cycles^12^, including the highest (i.e., ~300 ppmv) atmospheric CO_2_ concentrations recorded in ice cores^13^. Situated in an intermediate position between the temperate and tropical climate systems of the Northern Hemisphere (NH), and located at the moisture boundary separating the wetter western from the drier eastern Mediterranean region, the Tenaghi Philippon site is exceptionally sensitive to abrupt climate change^11,14^. This is illustrated by the prompt response of tree populations in the Tenaghi Philippon area to even subtle climatic forcing as shown by pollen records for both glacial^15–17^ and interglacial^18–20^ periods. Tenaghi Philippon was not a glacial refugium for trees in contrast to more humid intermontane locations^21^ such as Ioannina^22^ and Lake Ohrid^23^ from which vegetation records spanning multiple glacial-interglacial cycles have also been recovered. Hence, our record from Tenaghi Philippon is representative for vegetation biomes that are close to their lower moisture-tolerance limits such as the ‘subtropical dry forest’ biome that extends across c. 76 million ha in the Mediterranean region (Supplementary Fig. 1)^2^. Importantly, because our datasets are from a single site rather than being spliced from multiple sites with distinctly different environmental boundary conditions, they yield an internally consistent signal of climatic and ecological change.

## Results and discussion

### Vegetation dynamics at Tenaghi Philippon over the past 500 kyrs

**Fig. 1:**
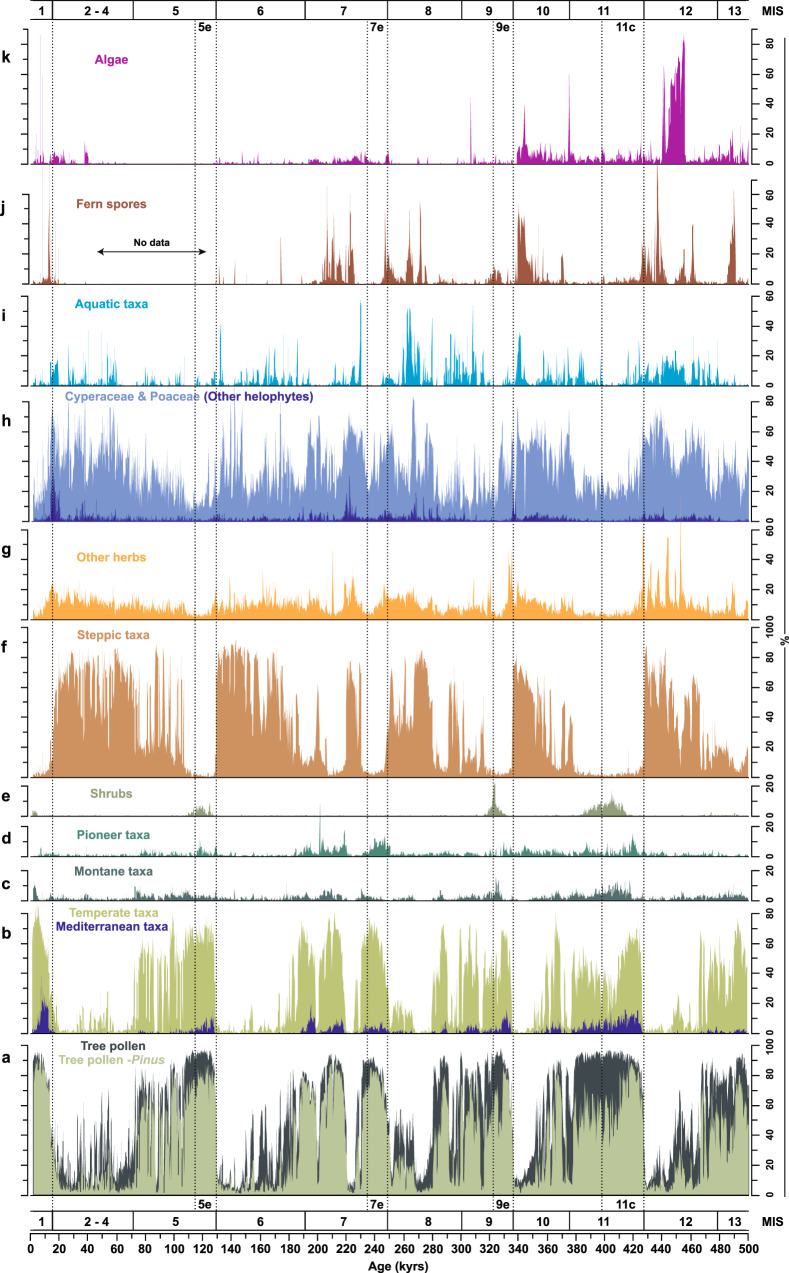
Overview of the pollen record from Tenaghi Philippon spanning the past 500 kyrs. **a** Tree-pollen percentages including (dark green) and excluding *Pinus* (olive green). **b** Temperate (light green) and Mediterranean (purple) tree taxa. **c** Montane tree taxa. **d** Pioneer tree taxa. **e** Shrubs. **f** Steppic taxa. **g** Other herbs. **h** Cyperaceae and Poaceae (light blue) and helophytes (dark blue). **i** Aquatic taxa. **j** Fern spores. **k** Algae. Marine Isotope Stages (MIS) adapted from ref. ^68^, and warmest interglacial sub-stages (vertical dashed lines) after ref. ^24^.

Our palynological results document two dominant alternating vegetation regimes at Tenaghi Philippon under the full spectrum of climatic boundary conditions realized during the past 500 kyrs: an open vegetation dominated by steppe biomes (tree-pollen <35 %) during glacials and forest biomes (tree-pollen >65 %) during interglacials (Fig. 1). While the transitions from glacials to interglacials are persistently marked by rapid reorganizations from steppe to forest biomes, the transitions from interglacials to glacials are highly dynamic and exhibit increased frequencies of abrupt millennial-scale shifts between forest and steppe biomes. Our high-resolution dataset documents that abrupt shifts in vegetation regime at Tenaghi Philippon were not confined to the last G/I cycle^15,19^, but are a persistent feature of the past four G/I cycles. While the duration of the regime shifts is persistently <200 years across the record, our palynological data from core intervals with higher-than-average temporal resolution indicate that these shifts actually occur on decadal scales.

**Fig. 2:**
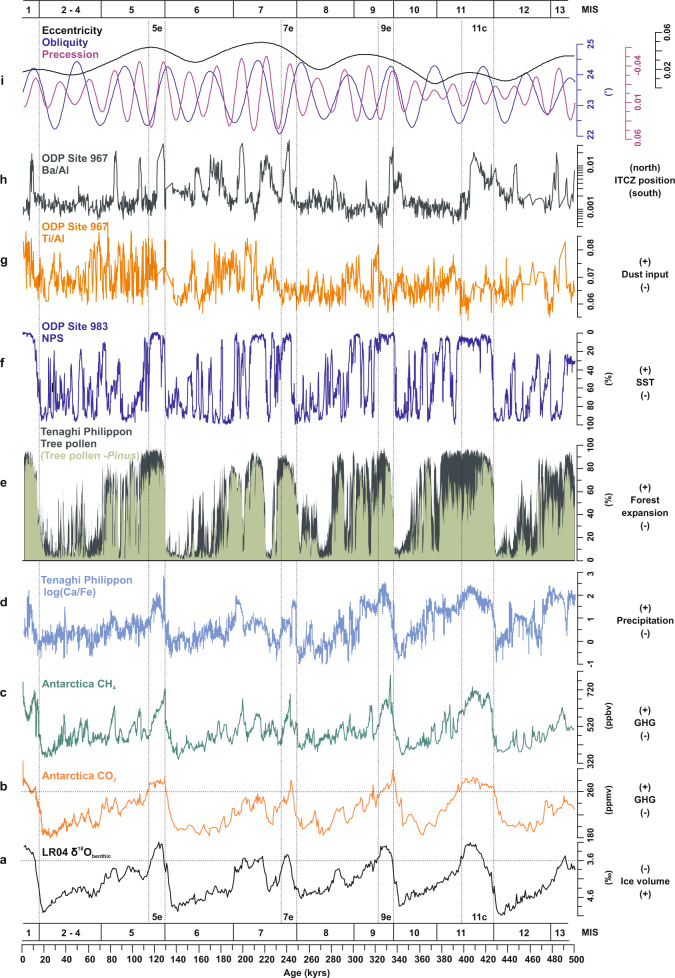
Vegetation and precipitation data from Tenaghi Philippon and selected climate records for the past 500 kyrs. **a** LR04 benthic δ^18^Ο stack^68^. **b** and **c** atmospheric CH_4_ (ref. ^31^) and CO_2_ (ref. ^13^) concentrations from Antarctic ice cores, respectively. **d** log(Ca/Fe) and **e** tree-pollen percentages from Tenaghi Philippon including (dark green) and excluding *Pinus* (light green). **f** *Neogloboquadrina pachyderma* ‘sinistral’ (NPS) percentages from ODP Site 983 (ref. ^32^). **g** and **h** Ti/Al and Ba/Al ratios from ODP Site 967, respectively (ref. ^33^). **i** Orbital parameters^34^. Horizontal dashed lines for the LR04 benthic δ^18^Ο stack (at 3.6 ‰) and the CO_2_ concentration in Antarctic ice cores (at 260 ppmv) represent threshold values for interglacial conditions^12^. Marine Isotope Stage (MIS) boundaries adapted from ref. ^68^, boundaries of warmest sub-stages (vertical dashed lines) of interglacials are from ref. ^24^. GHG greenhouse-gas concentrations, ITCZ Intertropical Convergence Zone, SST sea-surface temperature.

A pervasive characteristic of the terminal forest stages is the expansion of drought-tolerant, pine-dominated biomes coeval with the decrease of cold-tolerant, but drought-sensitive montane and shrub biomes (Fig. 1). This structural change within forest biomes occurred across all past four interglacials, despite the fact that each of these interglacials exhibits a unique floristic composition^11,14^. Based on our vegetation-independent age model, the demise of these drought-tolerant forest biomes persistently postdates the end of the warmest interglacial sub-stages (such as Marine Isotope Stages [MIS] 5e, 7e, 9e, and 11c) by about 5 to 20 kyrs (Supplementary Table 1) as determined in global temperature-proxy datasets such as the LR04 benthic δ^18^Ο stack and Antarctic ice-core records^12,24^ (Fig. 2), as well as in regional Mediterranean climate records^25^. The constant lag of the forest demise at Tenaghi Philippon with regard to global temperature-proxy datasets at times of glacial build-up implies a strong resilience of Mediterranean forest biomes to temperature decrease. This is supported by the continuous presence of cold-sensitive temperate and Mediterranean tree taxa beyond the end of the warmest interglacial sub-stages (Fig. 1). On this basis, the resilience of Mediterranean forest biomes across glacial inceptions appears to be determined by moisture availability^15,26^. However, validation of this hypothesis has yet been precluded by the absence of paired high-resolution (i.e., decadal- to centennial-scale) precipitation and vegetation proxy-record timeseries from the same locality.

To this end, we evaluate the log(Ca/Fe) fluctuations in the Tenaghi Philippon cores as an independent proxy for local precipitation. As the minerogenic content of the peat at Tenaghi Philippon is connected to an extensive karstic system in the mountain ranges surrounding the site, increased precipitation results in high Ca and low Fe contents in the Tenaghi Philippon sediments and vice versa (Methods). The log(Ca/Fe) values peak during interglacial maxima, indicating maximum precipitation at these times, and progressively decrease as global climate evolves gradually towards larger ice-sheets, colder conditions and lower greenhouse-gas concentrations of glacials (Fig. 2; Supplementary Fig. 2). The application of rolling correlations between the log(Ca/Fe) dataset from Tenaghi Philippon and the CO_2_ and CH_4_ records from Antarctic ice cores yields strong correlations between the records during peak interglacial conditions, whereas weak correlations prevail during the terminal interglacial phases (Supplementary Fig. 3). While this demonstrates that the hydrological record from Tenaghi Philippon responds sensitively to global climate dynamics, it also implies a systematic decoupling between the gradual moisture decrease and the abrupt forest contractions at times of glacial inception (Fig. 2).

### Assessing causal associations using convergent cross-mapping

To validate the hypothesis of a causal association between vegetation-regime shifts and hydroclimate change, we apply convergent cross-mapping (CCM) that can resolve causalities in complex systems with only partially coupled variables^27^, hence overcoming the shortcomings of simple correlation methods. Specifically, the CCM analysis allows to distinguish between direct and indirect causation from simple correlation between timeseries by assessing the extent to which an affected variable reliably estimates states of a causal variable (termed ‘CCM skill’) (ref. ^27^). In other words, if a variable A can be predicted using values of variable B, then we conclude that variable A is causally affecting variable B (Methods). As the cross-mapped estimates improve in ‘CCM skill’ with the length of the timeseries^27^, our high-resolution palynological and geochemical datasets are exceptionally well suited for this approach.

**Fig. 3:**
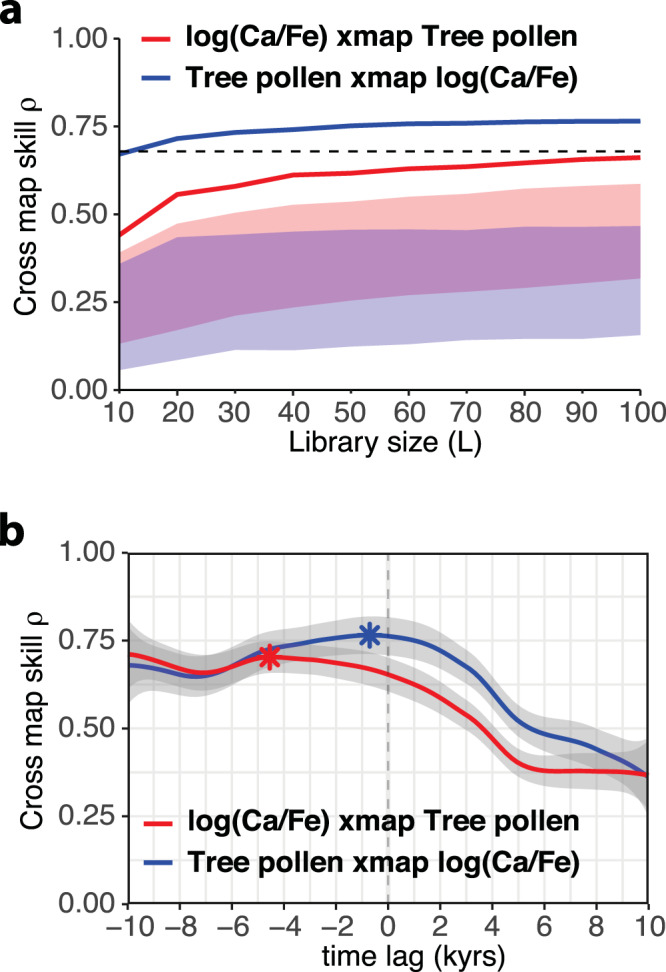
Convergent cross-mapping of the precipitation [log(Ca/Fe)] and vegetation (tree-pollen) datasets from Tenaghi Philippon. **a** Correlation of cross-mapped against observed values (‘CCM skill’) as a function of the timeseries (library) length ‘L’. The blue line represents the effect of log(Ca/Fe) on tree-pollen, whereas the red line represents the effect of tree-pollen on log(Ca/Fe). Both lines are above the blue and red shaded areas, which represent the 5th to 95th percentiles of the ‘CCM skill’ for 100 surrogate timeseries from null models, suggesting that convergence is significant for both pairs. As higher ‘CCM skill’ indicates stronger causality (Methods), the CCM analysis demonstrates a strong bidirectional coupling between precipitation [log(Ca/Fe)] and forest cover [tree-pollen] at Tenaghi Philippon. The black dashed line represents the cross-correlation of the two timeseries. **b** Time displacements maximizing CCM skill corresponding to the causal relationship between log(Ca/Fe) and tree-pollen (marked with stars). As the maximum ‘CCM skill’ of the effect of log(Ca/Fe) on tree-pollen is negative and closer to 0 (blue line) than the effect of tree-pollen on log(Ca/Fe) (red line), the analysis suggests a real causal and rapid effect of precipitation change on forest cover. Shaded areas represent the 95% confidence interval for predictions from the smoothing model.

Our results reveal increasing ‘CCM skills’ for the tree-pollen and log(Ca/Fe) datasets from Tenaghi Philippon (Fig. 3), suggesting a significant causal association between forest dynamics and precipitation. To confirm this unidirectional causality from bidirectional causality, which is often related to synchrony caused by forcing from a third independent factor, we evaluate the optimal time displacements of the ‘CCM skills’ (refs. ^28,29^) (Methods). Optimal time displacement means that we test the ‘CCM skill’ between tree-pollen and log(Ca/Fe) for different time lags. In that way, we can distinguish true causal associations from simple correlations and explore at which time lag the causal relationship between the two variables is strongest. Despite the bidirectional coupling between the tree-pollen and log(Ca/Fe) datasets as derived by the ‘CCM skills’, the peak for CCM estimates of the tree-pollen timeseries based on the log(Ca/Fe) timeseries, i.e., the effect of log(Ca/Fe) on tree-pollen, has an optimal lead of ~4–5 kyrs behind that for estimates of tree-pollen timeseries based on the log(Ca/Fe) timeseries (Fig. 3; Supplementary Table 2). Hence, the response of tree-pollen to log(Ca/Fe) change is significantly faster (<1 kyrs) than the response of log(Ca/Fe) to changes in tree-pollen (>4.5 kyrs). Collectively, the strong causality between the two timeseries and the rapid response of the tree-pollen signal to the log(Ca/Fe) variability testify that the abrupt vegetation-regime shifts at Tenaghi Philippon are determined by a crossing of precipitation thresholds.

While variations in atmospheric CH_4_ content, high-northern-latitude temperature, and the position of the Intertropical Convergence Zone (ITCZ) are considered the main driving forces of precipitation variability in the Mediterranean region over the past 500 kyrs^25,26,30^, it remains unclear whether these processes are pertinent to the projected precipitation shifts resulting from anthropogenic CO_2_ release^5^. In addition, any influence of these processes on vegetation dynamics other than through the modulation of moisture availability remains largely unexplored. To identify which of these climatic processes may play a critical role in near-future climate scenarios, we applied the CCM analysis to the log(Ca/Fe) and tree-pollen datasets from Tenaghi Philippon and representative climate-proxy timeseries, including global atmospheric greenhouse-gas (CO_2_ and CH_4_) concentrations from Antarctic ice cores^13,31^, *Neogloboquadrina pachyderma* ‘sinistral’ (NPS) abundances from Ocean Drilling Program (ODP) Site 983 in the North Atlantic as an indicator for high-latitude surface-ocean temperature^32^, and the Ti/Al and Ba/Al records from ODP Sites 967/968 as indicators for dust input and sapropel formation in the Eastern Mediterranean Sea associated with low-latitude migration of the ITCZ over Africa^33^. Moreover, we included orbital precession and obliquity^34^ in our analysis, although orbital configuration affects climate dynamics on millennial timescales and thus has an insignificant role in near-future climate change^12^. Our working hypothesis is that if orbital configuration is the predominant force of moisture variability and vegetation dynamics at Tenaghi Philippon over the past 500 kyrs, it can be assumed that Mediterranean forest biomes will be marginally affected by CO_2_-induced climate change in the future. To achieve a mechanistic understanding of the interacting processes, we further assessed the optimal time displacements of the ‘CCM skills’ to distinguish true causal associations from simple correlations and differentiate direct from indirect causality^28^ (Supplementary Table 2; Supplementary Fig. 4). The chronological limitations inherent in all the investigated climate-proxy timeseries may bear an effect on the statistical output. Hence, to explore potential effects of age-model uncertainties on the statistical results we also ran the ‘CCM’ analysis for all the timeseries pairs considering the maximum error range by shifting the Tenaghi Philippon chronology by +/− 5 kyrs (Supplementary Fig. 5).

**Fig. 4:**
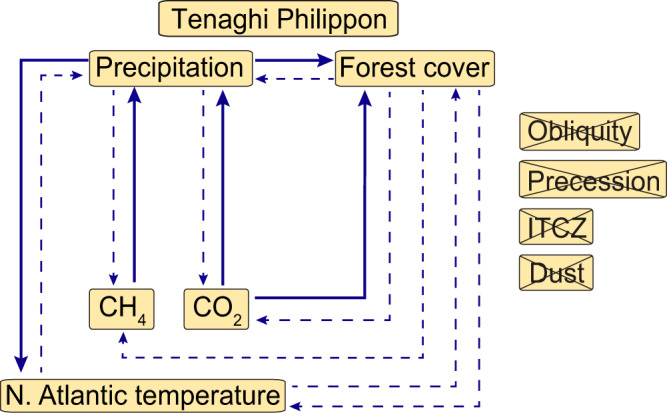
Summary of causal associations of the climate-proxy records as revealed by convergent cross-mapping (CCM) and time displacements of the ‘CCM skills’. Precipitation and forest cover at Tenaghi Philippon based on the log(Ca/Fe) and tree-pollen percentage datasets, respectively; orbital parameters from ref. ^34^ atmospheric CH_4_ (ref. ^31^) and CO_2_ (ref. ^13^) concentrations from Antarctic ice cores; position of the Intertropical Convergence Zone (ITCZ) and dust influx based on the Ba/Al and Ti/Al ratios from ODP Site 967, respectively (ref. ^33^); North Atlantic surface-water temperature based on *Neogloboquadrina pachyderma* ‘sinistral’ percentages from ODP Site 983 (ref. ^32^). Significance of causal associations was determined when ‘CCM skills’ of the proxy timeseries were outside the 5th and 95th percentiles computed from the randomly generated timeseries and the area under curve (AUC) value was higher than 0.8 (Supplementary Fig. 4; Supplementary Table 2). Bold arrows indicate significant causality (AUC > 0.9), whereas dashed arrows indicate weak causality (0.9>AUC > 0.8); crossed-out variables have no causal associations with the datasets from Tenaghi Philippon.

The CCM analysis yields a mixture of causal associations among the investigated variables, allowing to decipher the extent of high- and low-latitude climate forcing on precipitation and vegetation dynamics at Tenaghi Philippon. Specifically, the combined evaluation of the ‘CCM skills’ and optimal time displacements suggests that only the global atmospheric CO_2_ record has a causal effect on both the tree-pollen and precipitation records from Tenaghi Philippon (Fig. 4; Supplementary Fig. 4). Importantly, because the statistical output of the CCM runs remains stable even when accepting chronological errors of up to 5 kyrs, this effect appears to be independent of age-model uncertainties (Supplementary Fig. 5). Our results further document a strong causality between CH_4_ concentration and precipitation at Tenaghi Philippon, and no causal associations of the Tenaghi Philippon datasets with precession, obliquity, ITCZ migration, and dust. Again, these associations appear to be independent of age-model uncertainties. Our data further point to a causal association between precipitation at Tenaghi Philippon and North Atlantic temperature (as reflected by the log(Ca/Fe) and NPS timeseries, respectively; Supplementary Fig. 4); however, our sensitivity tests indicate a potential age-model effect on the ‘CCM skills’ (Supplementary Fig. 5), leading us to not further considering this correlation. Overall, it appears that only atmospheric CO_2_ variability has a rapid causal effect on vegetation dynamics at Tenaghi Philippon. Notably, this effect is realized by means of two discrete pathways. First, there is a direct causal effect of CO_2_ variability on vegetation (Fig. 4) through plant physiology processes^35^. Second, the causal associations document an indirect effect related to climatic processes: the CO_2_ variability dictates moisture change at Tenaghi Philippon (Fig. 4), which in turn exerts control on vegetation dynamics (Figs. 3 and 4). Collectively, the CCM analysis suggests an important effect of global atmospheric CO_2_ variability on Mediterranean forest biomes during multiple G/I cycles of the Quaternary.

### Effect of atmospheric CO_2_ on biome shifts

**Fig. 5:**
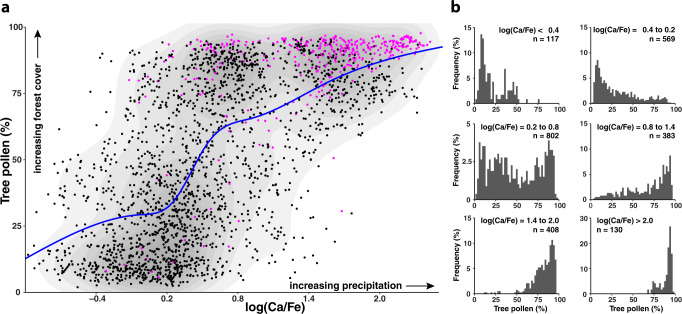
Forest cover as a function of precipitation at Tenaghi Philippon for the past 500 kyrs. **a** Cross-plot of tree-pollen percentages and log(Ca/Fe) values illustrating the persistence of two distinct and stable vegetation regimes for a wide range of precipitation levels. Rather than a gradual increase in tree cover with increasing precipitation at Tenaghi Philippon, non-linear regime shifts between steppe (low tree-pollen %) and forest (high tree-pollen %) biomes occur above a precipitation threshold (as suggested by the step-like blue line from a generalized additive model [GAM] fit). Purple circles show values at periods of atmospheric CO_2_ concentrations above 260 ppmv (ref. ^13^) representing interglacial conditions^12^. The gray shaded areas show the density of the data points (darker color for higher density). **b** Histograms of tree-pollen percentages at different log(Ca/Fe) values demonstrating a bimodal distribution with steppe biomes (low tree-pollen %) prevailing below a value of ~0.2 in log(Ca/Fe) and forest biomes (high tree-pollen %) dominating above a value of ~0.8. Regime shifts between the two biomes occur at a threshold value of ~0.5 in log(Ca/Fe).

The causality between the gradual variability in atmospheric CO_2_ concentration and the abrupt vegetation-regime shifts at Tenaghi Philippon over the past 500 kyrs attests to the crossing of critical thresholds that determine the state of Mediterranean forest biomes that are close to their lower moisture-tolerance limits. Cross-plot analysis of the tree-pollen and log(Ca/Fe) datasets reveal a non-linear relationship of the two timeseries, unequivocally demonstrating a bimodal distribution of tree-pollen percentages for a wide range of precipitation levels. Forest biomes prevail above a value of 0.8 in the log(Ca/Fe) record, whereas steppe biomes dominate below a log(Ca/Fe) value of 0.25 (Fig. 5). Our analysis shows that shifts between the two distinct vegetation regimes occur at a threshold value of ~0.5 in log(Ca/Fe). We explain this to represent the ~400 mm/year boundary in annual precipitation that is required for subtropical dry forest biomes to thrive in the Mediterranean region^1,2^. The results further indicate that both forest and steppe biomes can occur within a range of ±0.3 from the log(Ca/Fe) threshold (Fig. 5). This effect portrays vegetation multiple equilibria with prevailing climate conditions related to migration processes, longevity, and community dynamics^36^. On balance, our results demonstrate that regime shifts from forest to steppe biomes over the past 500 kyrs have occurred abruptly with the crossing of a precipitation threshold rather than as a gradual decrease in forest cover with precipitation. Most notably, peak precipitation levels and maximum forest cover at Tenaghi Philippon have prevailed persistently under CO_2_ concentrations above 260 ppmv as they are realized during full interglacial conditions^12^ (Fig. 5). Consequently, our analysis highlights that a precise determination of when forests at Tenaghi Philippon lost resilience within the natural CO_2_ range in the past can be achieved by examining the precipitation deficit as portrayed in the decadal-scale-resolution log(Ca/Fe) geochemical data.

Owing to the anthropogenic CO_2_ increase to non-analog levels of the past 500 kyrs, it remains unclear whether the relationship of atmospheric CO_2,_ precipitation and vegetation dynamics as realized during the past four G/I cycles at Tenaghi Philippon can be employed to forecast near-future vegetation-regime shifts in the Mediterranean region. Plants can acclimate rapidly to increasing CO_2_ through the reduction of the diffusive stomatal conductance of their leaves until they reach the limits of their phenotypic plasticity, which is twice as high as the CO_2_ concentrations predicted for the next century^37^. Moreover, higher CO_2_ concentrations may allow plants to maintain carbon uptake with lower stomatal conductance and, to some extent, help alleviate drought stress^38^. As plant physiology hence can compensate to a certain degree the moisture decrease under increasing CO_2_, determining the lower limit of moisture availability for maintaining forest resilience is the most critical factor for assessing future ecological stress in the Mediterranean region. Despite the non-analog states between the Quaternary and future CO_2_ concentrations, our paired palynological and geochemical record from Tenaghi Philippon can provide an indication for the minimum amount of precipitation required to preserve subtropical dry forest biomes in the Mediterranean region. At Tenaghi Philippon, the warmest interglacial conditions of the past 500 kyrs are persistently marked by log(Ca/Fe) values above 1.5 (Supplementary Fig. 2), and forest biomes are only maintained above a threshold value of 0.8 in the log(Ca/Fe) ratio (Fig. 5). Hence, a minimum decrease in annual precipitation of 40–45 % from full interglacial levels could set off a regime shift from forest to steppe biomes under natural CO_2_ concentrations. Climate scenarios for a CO_2_-induced temperature rise of 2 °C relative to present in the coming decades predict a decrease of up to 30 % in annual precipitation in the Mediterranean region^5^. This adds up to the natural reduction of water availability in the Mediterranean region in comparison to the wettest interval of the Holocene^39^. Climate scenarios also predict decreased surface runoff and summer soil moisture in the Mediterranean region during the 21st century^40^. Specifically, the water deficiency will stem from an increase in canopy water consumption with additional vegetation growth along with longer and warmer growing seasons, despite the increased surface resistance to evapotranspiration and vegetation total water use efficiency^40^. Against the background of declining resilience of arid forest biomes to climate variability over the past three decades^41,42^, the combined natural and human-induced water deficit in the near-future along with the increasing human pressure on Mediterranean ecosystems^43^ raises concerns for the crossing of the moisture threshold required for the persistence of subtropical dry forest biomes. Our 500-kyr-long record from Tenaghi Philippon indicates that an abrupt collapse of forest communities and a rapid switch into a steppe environment would almost be inevitable, urging for sustainable conservation of Mediterranean forests to bolster their resilience to near-future climate change.

## Methods

### Study site and regional setting

The sedimentary archive preserved in the subsurface of the Tenaghi Philippon peatland in the Drama Basin of NW Greece (see ref. ^11^ for an in-depth review) yields a key terrestrial archive for deciphering global climate dynamics^12,44^. The Tenaghi Philippon peatland is situated at an elevation of c. 40 m above sea level surrounded by mountain ranges with altitudes of up to 2232 m (Supplementary Fig. 1). From the early Pleistocene onwards, limnotelmatic conditions prevailed across large parts of the Drama Basin, resulting in a c. 172-m-thick, stratigraphically continuous succession in the southeastern part of the basin that is overwhelmingly composed of fen peat; this succession represents the thickest known peat deposit worldwide^11,45^.

Climatically, Tenaghi Philippon is characterized by a typical Mediterranean seasonal regime modified by continental influence associated with lower winter temperatures and wetter summers as they are characteristic for the NE Mediterranean region. Meteorological data from the nearby town of Drama (Supplementary Fig. 1) record a mean annual temperature of 16.1 °C, and mean July and January temperatures of 27.2 and 5.2 °C, respectively, for the period 1975–2008 (ref. ^46^). Owing to the region’s pronounced topography, katabatic flow of cold air masses from the surrounding mountains into the Drama Basin plain causes much lower winter temperatures on the basin floor than at comparable elevations along the nearby coast^47^. Winter temperature is also influenced by anomalously cold and windy episodes connected to southward outbreaks of polar air masses from the Russian High^48^ often causing the development of frost. Modern precipitation in the Drama Basin is connected to the penetration of westerly storm tracks across southern Europe and Mediterranean cyclogenesis (ref. ^49^ and references therein). Precipitation in the basin shows a strong seasonal distribution with a maximum in autumn and winter, and moderately dry summers. Based on the Drama meteorological station, mean annual precipitation is 520 mm, with August and September being the driest (mean: 28 and 29 mm, respectively) and December (mean: 67 mm) the wettest months; 40 % of the annual precipitation occur from November to February, and only 25 % occur from June to August (period 1975–2008; ref. ^46^). The annual precipitation in the surrounding mountain ranges is up to 1100 mm (period 1966–1991; ref. ^50^) and thus twice as high as in the basin itself. A 100 m increase in elevation is associated with a precipitation increase between ~30 and ~55 mm/year in the western and eastern mountain ranges, respectively^50^.

Owing to its complex geomorphology and broad range of climatic conditions, the Drama Basin and its surroundings are home to a highly diverse flora^51–54^ comprising four main ecozones, i.e., temperate mountain systems, temperate continental forests, subtropical mountain systems, and subtropical dry forests^2^ (Supplementary Fig. 1). Dominant conifer trees are *Abies alba* Mill. and *Pinus nigra* J.F. Arnold, whereas the most common deciduous trees include *Acer* spp., *Carpinus orientalis* Mill., *Castanea sativa* Mill.*, Cornus mas* L., *Corylus avellana* L., *Crataegus monogyna* Jacq., *Fagus sylvatica* L., *Ostrya carpinifolia* Scop., *Quercus frainetto* Ten., *Q. pubescens* Willd., *Q. petraea* (Matt.) Liebl., and *Tilia tomentosa* Moench. Sclerophyllous evergreen shrubs and trees comprise *Arbutus unedo* L., *Erica arborea* L., *Juniperus* sp., *Pistacia terebinthus* L., *Quercus coccifera* L., and *Q. ilex* L. Noteworthy, the mountain ranges surrounding the Drama Basin harbor the Balkan endemic trees *Abies borissii-regis* Mattf.*, Acer heldreichii* Boiss.*, Acer hyrcanum* Fisch. & C.A. Mey.*, Pinus peuce* Griseb., *Salix xanthicola* K. I. Chr. as well as the southernmost occurrences of *Betula pendula* Roth and *Picea abies* (L.) H. Karst. on the Balkan Peninsula^54^.

### Tenaghi Philippon sediment cores

The study is based on a composite sequence developed by the alignment of two parallel drillcores retrieved in a distance of 50 meters from each other (TP-2005 Phi1 and Phi2 cores; 40° 58’ 24.0” N, 24° 13’ 25.2” E) and one drillcore (TP-2009; 40° 57’ 39.5” N, 24° 16’ 03.1” E) retrieved 4.4 km south from the TP-2005 core location (Supplementary Fig. 1). Correlations between the cores were established based on a set of independent proxies, i.e., cryptotephra identification^55^, X-ray fluorescence (XRF) core-scanning data (Supplementary Fig. 6), and palynostratigraphy.

We here present information from the interval 4–91 m composite depth of the Tenaghi Philippon archive spanning the past c. 500 kyrs continuously (see ‘Chronology’). The uppermost 4 m of the composite record have been excluded from this study because the peat sequence is disturbed by anthropogenic activities related to the drainage of the peatland and agriculture. Lithologically, the succession consists predominantly of fen pet. Specifically, the intervals 4.0–58.5 m and 67.0–82.0 m are made up of fen peat, the intervals 58.5–67.0 m and 84.0–91.0 are dominated by organic-rich mud, and the interval 82.5–84.0 m consists of lake marl.

### Peat geochemistry

The geochemical composition of the inorganic components in the Tenaghi Philippon peat cores is predominantly influenced by mineral-rich groundwater and, to a lesser extent, by spring and surface waters flowing into the Drama Basin from the surrounding mountain ranges^45,56^. The subsurface of the Drama Basin contains a shallow unconfined aquifer with a depth of ~1.5 m at Tenaghi Philippon^45,50^. Based on hydrological investigations, the groundwater recharge predominantly occurs from carbonate terrains (i.e., limestones and marbles) in the surrounding mountain ranges (Supplementary Fig. 1) accounting for >50 % of the total precipitation^50^. Rainwater from the surrounding mountains reaches the aquifer in the Drama Basin within few weeks^50^. To a lesser extent, groundwater recharge also occurs within the Drama Basin due to the positive precipitation/evapotranspiration balance^50^ even during drier-than-average conditions as depicted in meteorological data since 1975 (ref. ^46^). As such, the groundwater in the Drama Basin is strongly enriched in Ca as a result of carbonate dissolution. In addition, relatively high Fe and Si concentrations are contained in the groundwater at the basin margins originating from alluvial fan sediments deposited close to the mountain ranges^50,56^. Considering that a dominance of mechanical over chemical erosion of carbonates in the Eastern Mediterranean region only emerges under hyper-arid conditions at a mean annual precipitation <200 mm (ref. ^57^), the carbonate content in the drillcores from the Drama Basin can be firmly attributed to result from carbonate dissolution related to the activity of the karst system in the surrounding mountain ranges.

Detailed coal petrographic and geochemical characterization of the uppermost ~10 m of the Tenaghi Philippon peatland suggests distinct differences in inorganic-geochemical composition in the peat deposited during the Last Glacial *versus* that deposited during the Holocene^58–60^: the Holocene peat is rich in Ca, whereas peat from the Last Glacial is depleted in Ca, but enriched in Fe, K, Ti, and Si; moreover, the degree of peat humification and gelification of the organic matter, which is enhanced under low-oxygen conditions and during periods of high water content in the sediments^61^, is high in the Holocene and low in the Last Glacial sediments. In addition, mineralogical analysis across the Last Interglacial interval of the core material shows highest contents of Ca-bearing minerals in the peat during the time of sapropel formation in the Eastern Mediterranean Sea, which is related to increased precipitation in the northern Mediterranean borderlands^25,62,63^. Highest contents of siliciclastics are registered during the transition phase from full interglacial conditions to the subsequent glacial, resulting from a higher erosion of surface sediments due to increased forest openness^64^. Together, these results suggest that (i) increased precipitation during interglacials leads to a highly active karstic system, which in turn results in a high groundwater table, enhanced telmatic conditions at Tenaghi Philippon, and high Ca content of the peat; (ii) decreased precipitation during glacials leads to a less active karstic system causing a lowering of the groundwater table, and enhanced siliciclastic sedimentation from the basin and the peatland-exposed margins related to surface soil erosion due to low vegetation cover.

To confirm the observation from the uppermost ~10 m of the core material that the interglacial peat at Tenaghi Philippon is enriched in Ca, whereas the glacial peat is enriched in Fe, K, Ti, and Si, we examined the variability in elemental composition over the past four G/I cycles by applying a PCA analysis to the elemental dataset derived by XRF core scanning. The PCA yields two major components that account for 73.1% of the total variance of the elemental dataset (Supplementary Fig. 7). The first component (PC1) explains 55.7 % of the total variance and is marked by positive loadings of Al, Fe, K, Mn, Si, and Ti, and negative loadings of Ca and S. The second component (PC2) explains 17.4 % of the total variance. Its positive pole is primarily driven by S and Fe, and to a lesser extent by Ti, whereas its negative pole is primarily driven by Ca and Mn. The two principal components can be explained to differentiate between intervals of high groundwater table rich in carbonates (negative scores in both PCs) and intervals of low groundwater table rich in siliciclastics due to enhanced clastic sedimentation from the basin margins (positive scores in PC1) and in sulfur due to organic matter decomposition under anaerobic conditions (positive scores in PC2). In light of the significance of Ca and Fe in reconstructing karstic system dynamics at Tenaghi Philippon, and considering that these elements yield the highest XRF counts throughout the studied sequence, we use the log(Ca/Fe) ratio as an indicator of moisture variability with high(low) values corresponding to high(low) groundwater table and, by extension, high(low) precipitation.

### Chronology

The age model is based on combined acceleration mass spectrometry (AMS) ^14^C dating, tephrochronological and cyclostratigraphic techniques. Firstly, 67 available ^14^C dates^65,66^ and ten independently dated (crypto)tephra layers out of a total of 57 detected in the uppermost 91 m composite depth of the Tenaghi Philippon cores^55,65,67^ were employed to establish chronostratigraphic anchor points and calculate average sedimentation rates. Subsequently, cyclostratigraphic analysis was performed on the log(Ca/Fe) record, which yielded pronounced cyclic variability in the 12–16 m range and an additional, albeit minor, cycle within the 5–6 m period band (Supplementary Figs. 8 and 9). Considering an average sedimentation rate of ~15 cm/kyr for the Philippi peatland during the past 500 kyrs, the 12–16 m cycle corresponds to a period of 80–106 kyrs, and the 5–6 m cycle to a period of 33–40 kyrs. These periodicities are strongly reminiscent of orbital (short) eccentricity and obliquity, respectively (ref. ^34^), suggesting that the cycles in the log(Ca/Fe) record represent G/I variability paced by orbital eccentricity during the Middle and Late Pleistocene^44,68^, with interglacials corresponding to high log(Ca/Fe) values and vice versa (see also ‘Peat geochemistry’).

Subsequently, a continuous chronology was developed by tuning the XRF-based log(Ca/Fe) record to the ice-sheet model of ref. ^69^, assigning maxima (minima) in the log(Ca/Fe) record to minima (maxima) in global ice volume (Supplementary Fig. 9). The tuning approach presupposes an in-phase behavior between changes in the log(Ca/Fe) record and the moisture budget at Tenaghi Philippon during G/I cycles. This assumption is confirmed by the in-phase relationship between the log(Ca/Fe) record and the ice-sheet model during the last ~40 kyrs (corresponding to the uppermost 13 m in the Tenaghi Philippon sequence), which is robustly dated by ^14^C and tephrochronology^65,66^. Considering the error estimates of the ice-sheet model towards radiometric ages for the past ~650 kyrs^69^, the age-model uncertainties for the Tenaghi Philippon chronology are <5 kyrs. The robustness of the age model is confirmed by the independently dated (crypto)tephra layers detected in the Tenaghi Philippon cores^55,65,67^ all of which fall within the 5-kyrs error uncertainty (Supplementary Fig. 9).

### Palynological analysis

Our study is based on 2347 palynological samples from the 4–91 m composite core depth interval of the Tenaghi Philippon archive. Sampling resolution is between 2 and 6 cm, which based on the age model for the composite core yields a mean temporal resolution of 209 years. For the core intervals corresponding to MIS 6–7 and MIS 9–13, a total of 1183 new samples were counted. For MIS 1–5 and MIS 8, our analysis is based on the raw data of ref. ^15,17,19^ that were augmented by additional pollen counts in order to increase their temporal resolution and statistical fidelity.

Palynological preparation comprised sediment freeze-drying, weighing, spiking with *Lycopodium* spores, treatment with HCl (10 %), NaOH (10 %), HF (when necessary; 40 %), heavy-liquid separation with Na_2_WO_4_ x 2H_2_O (when necessary), acetolysis, sieving through a 7 μm mesh, and slide preparation using glycerine jelly. Between 53 and 3661 (mean: 500) pollen grains were counted per sample excluding pollen from aquatic plants and fern spores. Pollen percentages were calculated on the number of pollen grains from terrestrial plants excluding Cyperaceae and Poaceae because of their natural over-representation at Tenaghi Philippon^14^. The percentages of Cyperaceae and Poaceae, aquatic taxa, fern spores and algae were calculated on the basis of the main sum plus the counts for the individual taxa.

### X-ray florescence core scanning

The TP-2005 and TP-2009 cores were scanned with an AVAATECH (GEN-4) XRF core scanner equipped with an OXFORD ‘Neptune 5200’ series 100 W X-ray source with a Rhodium anode at the Institute of Earth Sciences, Heidelberg University. Prior to scanning, all core sections were warmed to room temperature, and the core surfaces were smoothed and covered with 4-μm-thick Ultralene® foil to avoid contamination of the detector window and desiccation of the cores. Scanning was carried out at a 10 kV energy level without filter using a current of 200 mA, a counting time of 10 s, and slit sizes of 5 mm downcore and 5 or 10 mm crosscore. Data acquisition was via a RAYSPEC SiriusSD 65 mm^2^ Silicon Drift Detector (Model 878-0616B), and a BRIGHTSPEC Topaz-X Multichannel Analyzer. Processing of the X-ray spectra was performed using the BRIGHTSPEC bAxilBatch software (Version 1.4). The individual element counts were normalized to the total counts of all processed elements for each measurement excluding Ag and Rh because these elements are biased by the signal generation as they are included in the beam collimator of the detector and the X-ray source, respectively. In addition, log elemental ratios were used to eliminate non-linear matrix effects and constant-sum constraints^70,71^. Based on the age model, the average temporal resolution of the XRF data is 32 years.

### Convergent cross-mapping

To examine potential causalities between the Tenaghi Philippon pollen and log(Ca/Fe) datasets and other paleoclimate proxy records, we applied convergent cross-mapping (CCM) that allows us to distinguish causality from simple correlation in multivariate timeseries of deterministic dynamical systems^27^. Representing a powerful method to infer causality among complex Earth system processes^72^, the CCM analysis has been successfully applied to recent ecological^73^ and climate timeseries^74^, as well as Antarctic paleoclimate datasets^28^. The CCM analysis is based on Takens’ Theorem^75,76^, which states that if timeseries variable A influences timeseries variable B in a dynamical system, then the state A(t) can be reliably predicted from the state of B(t). On this basis, CCM allows us to assess the extent to which the record of the affected variable B reliably estimates states of the causal variable A. This is achieved by comparing Pearson’s correlation coefficient (ρ) between the predicted and observed values of A(t) (termed ‘CCM skill’ after ref. ^27^). As convergence is used to distinguish between direct and indirect causation from simple correlation, the cross-mapped estimates improve in ‘CCM skill’ with the length of the timeseries that is used to predict A from B.

CCM was performed using the ccm function of the rEDM package (Version 1.2.3.; ref. ^77^) in R (Version 3.6.3; ref. ^78^). All datasets were interpolated linearly to equidistant 1 kyr time steps prior to CCM analysis. Optimal CCM prediction parameters (i.e., the combination of embedding dimension [*E*] and time delay [*τ*] from a range of *E* [1–10] and *τ* [1–4] that yielded the highest forecast skill *ρ*) are derived from simplex projection using the *simplex* function included in the rEDM package, utilizing the entire timeseries as both library and predictor variable. Significance was determined by cross-mapping timeseries A against 100 bootstrapped and randomly phase-shifted surrogate iterations of timeseries B, and vice versa^79^. The sensitivity of the method was assessed by calculating the ‘CCM skill’ for every timeseries against 100 surrogate timeseries, utilizing five combinations of *E* and *τ* that yielded the highest forecast skill ρ as derived from the simplex projection. The results were evaluated using receiver operating characteristics (ROC) curves and, more specifically the area under curve (AUC). Both ROC and AUC calculations were performed using the ROCR package (Version 1.0-11; ref. ^80^) in R (Version 3.6.3; ref. ^78^).

To determine the true direction of causality and to identify a potential occurrence of dynamic synchrony (i.e., bidirectional CCM convergence despite unidirectional causation)^75^ that frequently occurs in strongly coupled dynamic systems such as the Earth’s climate system, we also applied time-delayed CCM, employing a prediction horizon of 10 time steps (1 kyr each) in both directions^28,29^. This approach is based on the principle that in dynamic systems with delayed responses to extrinsic forcing, the optimal ‘CCM skill’ for a variable should occur with an asymmetrical time delay for both variables. In contrast, dynamic synchrony (i.e., variables A and B are forced by a third variable Z) should occur nearly instantaneous and with symmetrical time displacement for both variables^28,29^. The time displacements can be utilized further to reveal delayed onsets of cause and effect in dynamic systems. A maximum ‘CCM skill’ close to a time displacement of 0 (or to the embedding lag *τ*) implies a (nearly) instantaneous effect. The further the maximum ‘CCM skill’ diverges from 0, the slower is the response time of said effect. Negative time displacements that lead to higher CCM skill would imply a real causal effect as long as there is a difference in the optimal lag between the CCM skills of one variable to the other. In contrast, a positive time displacement would imply that the affected variable is better at predicting future values of the driving variable and can be used to rule out causality^29^. Time-delayed CCM was performed in R (Version 3.6.3) using the *ccm* function by setting the *tp* parameter to non-zero (e.g., ±10) (ref. ^29^). The time displacement yielding the maximum ‘CCM skill’ was determined by smoothing (*x* ~ *y*^2^, *n* = 100, span = 1) the ‘CCM skill’ of 100 bootstrapped library sets of size 100 as a function of time displacement (±10) (ref. ^28^). Values towards the limits of the time displacement were not considered due to high uncertainties of the smoothing function.

### Generalized additive models

Generalized additive models (GAM) is a statistical modeling technique utilizing non-linear smoothing functions to model non-linearities in datasets^81^. GAM values were calculated using the *gam* function from the *mgcv* package (Version 1.8-31; ref. ^82^) in R (Version 3.6.3; ref. ^78^) using cubic regression splines (*cr*) and a smoothing dimension of *k* = 25.

### Principal component analysis

PCA was applied to the elemental data derived through XRF core scanning in order to examine the relationships among selected elements with robust signals across the entire sequence (i.e., Al, Ca, Fe, K, Mn, P, S, Si, and Ti). The analysis was performed using the function *prcomp* implemented in the R base package (Version 3.6.3; ref. ^78^), which utilizes a singular value decomposition function to introduce unit variances.

### Rolling correlation

Rolling correlations were used to identify changes in the correlation between two times series through time. The method utilizes a rolling window in which the correlation is calculated independently of the surrounding data, with the adjusting window size yielding information on shorter (small window size) or longer (big window size) timeframes. The analysis was performed in R (Version 3.6.3; ref. ^78^) using the *surrogateCor* function from the *astrochron* package (Version 1.0; ref. ^83^), with window sizes ranging between 20 and 50 time steps, increasing in increments of 10, and with every time step representing 1 kyr.

### Spectral analysis

To identify orbital frequencies in the log(Ca/Fe) record we applied REDFIT analyses^84^ as implemented in PAST software. The significance levels at 80%, 90%, and 95% were tested using a Monte-Carlo simulation. To trace the spatial development of the identified dominant frequencies in the depth domain we further applied evolutive harmonic spectral analysis (EHA) using the multitaper method^85^ as implemented in the function *eha* in the *astrochron* R software package^83^. The log(Ca/Fe) dataset was resampled at 0.01 m levels prior to the application of the EHA.

## Supplementary information

Supplementary Information

## Data Availability

The pollen and XRF data generated in this study have been deposited in the PANGAEA database under accession codes (10.1594/PANGAEA.943592, 10.1594/PANGAEA.943593). Source data are provided with this paper.

## Source data

Source Data

## Code availability

The fully reproducible code for the convergent cross-mapping (CCM) analysis is available on ‘GitHub’(https://zenodo.org/badge/latestdoi/452267408).
