# Supplementary material for: Atmospheric CO2 forcing on Mediterranean biomes during the past 500 kyrs
Source: Nat Commun. 2023 Mar 25;14:1664. doi: 10.1038/s41467-023-37388-x (PMC10039881; doi:10.1038/s41467-023-37388-x)
Supplement: Supplementary file 1 — Supplementary Information [file 41467_2023_37388_MOESM1_ESM.pdf]

Supplementary Information for

‘Atmospheric CO_2 forcing on Mediterranean biomes during the past 500 kyrs’

Andreas Koutsodendris[1*], Vasilis Dakos[2,3], William J. Fletcher[4], Maria Knipping[5], Ulrich Kotthoff[6,7], Alice M. Milner[8], Ulrich C. Müller[9,10], Stefanie Kaboth-Bahr[1,11], Oliver A. Kern[1], Laurin Kolb[1], Polina Vakhrameeva[1], Sabine Wulf[12], Kimon Christanis[13], Gerhard Schmiedl[6], Jörg Pross[1,10]

1. Institute of Earth Sciences, Heidelberg University, Heidelberg, Germany
2. Institute des Sciences de l’Évolution, Université de Montpellier, CNRS, IRD, EPHE, Montpellier, France
3. Institut d’Écologie et des Sciences de l’Environnement de Paris (iEES Paris), Sorbonne Université, Paris, France
4. School of Environment, Education and Development, The University of Manchester, Manchester, United Kingdom
5. Department of Molecular Botany, Institute of Biology, University of Hohenheim, Stuttgart, Germany
6. Center for Earth System Research and Sustainability, Institute of Geology, Hamburg University, Hamburg, Germany
7. Leibniz Institute for the Analysis of Biodiversity Change (LIB), Hamburg, Germany
8. Department of Geography, Royal Holloway University of London, London, United Kingdom
9. Parlamentsstraße 32, 60385, Frankfurt am Main, Germany
10. Senckenberg Biodiversity and Climate Research Centre (SBiK-F), Senckenberg Gesellschaft für Naturforschung, Frankfurt am Main, Germany
11. Institute of Geosciences, University of Potsdam, Potsdam-Golm, Germany
12. School of the Environment, Geography and Geosciences, University of Portsmouth, Portsmouth, United Kingdom
13. Department of Geology, University of Patras, Rio, Greece

*Corresponding author; andreas.koutsodendris@geow.uni-heidelberg.de

Supplementary Table 1. Durations of forest phases at Tenaghi Philippon during the warmest interglacial substages of the past 500 kyrs and mean durations of the respective interglacial substages based on global marine and ice-core datasets[1]. The onset of interglacial forest phases at Tenaghi Philippon is defined at the mid-points between the minimum/maximum values in tree-pollen abundances before and after a pronounced decrease in steppic taxa abundances close to a glacial termination. The end of interglacial forest phases is defined at the mid-points between the maximum/minimum values in tree-pollen abundances closely before and after the first pronounced increase in steppic taxa abundances.

**Interglacials in marine and ice core datasets**[1]		**Tenaghi Philippon forest phases**	
Substage	Duration mean/median (kyrs)	Duration (kyrs)	Onset/end (kyrs)
MIS 1	12.1 / 12.0	13.0	13.0 – n.a
MIS 5e	17.4 / 15.8	22.2	128.7 – 106.4
MIS 7a-c	11.1 / 10.0	19.1	219.8 – 200.7
MIS 7e	6.5 / 5.1	18.0	247.9 – 229.9
MIS 9e	15.7 / 14.4	19.6	335.3 – 315.6
MIS 11c	27.7 / 26.9	49.1	426.7 – 377.6

Supplementary Table 2. Convergent cross-mapping (CCM) results for the Tenaghi Philippon and global proxy timeseries. CCM skills, correlation coefficients, areas under curve (AUC), and optimal time displacements between the tree-pollen (a, b) and the log(Ca/Fe) (c, d) timeseries, respectively, and orbital obliquity and precession[2], Antarctic ice-core atmospheric CH_4 (ref. 3) and CO_2 (ref. 4) concentrations, *Neogloboquadrina pachyderma* 'sinistral' abundances from Ocean Drilling Program (ODP) Site 983 (ref. 5), and XRF-based Ba/Al and Ti/Al ratios from ODP Site 967/968 (ref. 6). E: embedding dimension; τ: time delay. Tables are sorted based on the time displacement values. 'A xmap B' quantifies the causal effect of variable B on variable A by predicting the state of variable A from E lagged time-series fragments of variable B. Time displacements are negative when past values of the cross-mapped variable B (*cause*) are estimated by the embedded variable A (*effect*). Positive time displacements suggest that there is no flow of causal information from variable B (*cause*) to variable A (*effect*), and hence changes in variable B are not reflected in variable A until sometime in the future. Area under curve (AUC) of the sensitivity test results is a measure of performance across all possible threshold settings and can be interpreted as the probability that CCM predictions are ranked correctly (i.e., AUC = 1 represents a 100 % probability of correct predictions and AUC = 0 represents a 0 % probability of correct predictions). ρ: Pearson's correlation coefficient.

a)

**Variable A (effect)**		**Variable B (cause)**	**E**	**τ**	**CCM skill**	**ρ**	**AUC**	**Time displacement**
Tree-pollen	*xmap*	Obliquity	7	2	0.300	0.091	0.42	-7.778
Tree-pollen	*xmap*	NPS	7	2	0.636	-0.612	1.00	-2.727
Tree-pollen	*xmap*	log(Ca/Fe)	7	2	0.765	0.679	1.00	-0.707
Tree-pollen	*xmap*	CO_2	7	2	0.854	0.677	1.00	-0.303
Tree-pollen	*xmap*	CH_4	7	2	0.757	0.577	1.00	0.505
Tree-pollen	*xmap*	Precession	7	2	0.261	0.075	0.85	7.980
Tree-pollen	*xmap*	Ba/Al	7	2	0.178	0.068	0.69	7.980
Tree-pollen	*xmap*	Ti/Al	7	2	0.074	0.068	0.31	7.980

b)

**Variable A (effect)**		**Variable B (cause)**	**E**	**τ**	**CCM skill**	***ρ***	**AUC**	**Time displacement**
Precession	*xmap*	Tree-pollen	2	2	0.094	0.075	0.53	-7.778
Ba/Al	*xmap*	Tree-pollen	9	4	0.288	0.068	0.72	-7.778
CH_4	*xmap*	Tree-pollen	6	2	0.605	0.577	1.00	-4.747
log(Ca/Fe)	*xmap*	Tree-pollen	10	4	0.661	0.679	1.00	-4.545
CO_2	*xmap*	Tree-pollen	5	4	0.743	0.677	1.00	-4.343
NPS	*xmap*	Tree-pollen	6	2	0.658	-0.612	1.00	-4.141
Obliquity	*xmap*	Tree-pollen	2	2	0.066	0.091	0.20	7.980
Ti/Al	*xmap*	Tree-pollen	9	4	0.123	0.068	0.31	7.980

c)

**Variable A (effect)**		**Variable B (cause)**	**E**	**τ**	**CCM skill**	***ρ***	**AUC**	**Time displacement**
log(Ca/Fe)	*xmap*	Obliquity	10	4	0.530	0.098	0.53	-4.949
log(Ca/Fe)	*xmap*	Ba/Al	10	4	0.368	-0.007	0.82	-4.747
log(Ca/Fe)	*xmap*	Tree-pollen	10	4	0.661	0.679	1.00	-4.545
log(Ca/Fe)	*xmap*	Precession	10	4	0.551	-0.041	0.86	-4.343
log(Ca/Fe)	*xmap*	NPS	10	4	0.520	-0.489	0.97	-4.343
log(Ca/Fe)	*xmap*	CO_2	10	4	0.878	0.596	1.00	-0.505
log(Ca/Fe)	*xmap*	CH_4	10	4	0.755	0.529	1.00	-0.101
log(Ca/Fe)	*xmap*	Ti/Al	10	4	0.351	-0.036	0.94	1.111

d)

**Variable A (effect)**		**Variable B (cause)**	**E**	**τ**	**CCM skill**	***ρ***	**AUC**	**Time displacement**
Precession	*xmap*	log(Ca/Fe)	2	2	0.115	-0.041	0.68	-7.778
Ba/Al	*xmap*	log(Ca/Fe)	9	4	0.306	-0.007	0.69	-7.778
CH_4	*xmap*	log(Ca/Fe)	6	2	0.497	0.529	0.98	-5.152
CO_2	*xmap*	log(Ca/Fe)	5	4	0.656	0.596	1.00	-2.929
NPS	*xmap*	log(Ca/Fe)	6	2	0.562	-0.489	1.00	-1.111
Tree-pollen	*xmap*	log(Ca/Fe)	7	2	0.765	0.679	1.00	-0.707
Obliquity	*xmap*	log(Ca/Fe)	2	2	0.108	0.098	0.44	-0.303
Ti/Al	*xmap*	log(Ca/Fe)	9	4	0.259	-0.036	0.60	7.980

Supplementary Table 3. List of taxa included in the individual palynological groups shown in Figure 1. Taxa in brackets mark single or sporadic occurrences.

Group	Taxa
Algae	*Botryococcus* sp., *Coelastrum* sp., *Mougeotia* sp., *Pediastrum* sp., *Spirogyra*, Zygnemataceae undiff.
Aquatic taxa	*Alisma*, *Callitriche*, *Elatine*, *Eriocaulon aquaticum*, *Hottonia*, *Hydrocharis* type, Lemnaceae, *Littorella* type, *Menyanthes trifoliata, Myriophyllum* sp., *Nuphar*, *Nymphaea*, *Persicaria amphibia*, *Potamogeton*, *Sagittaria sagittifolia*, *Stratiotes aloides*, *Typha angustifolia/Sparganium* type, *Typha latifolia*
Helophytes	*Allium inundatum*, *Epipactis palustris*, *Filipendula*, *Ludwigia palustris*, *Lysimachia vulgaris*, *Lythrum*, *Parnassia palustris*, *Peplis*, Ranunculaceae undiff., *Ranunculus acris* type, *Rumex* undiff., *R. aquaticus*, *R. acetosa* type, *Utricularia*, *Thalictrum*
Mediterranean taxa	*Nerium oleander*, Oleaceae undiff., *Olea*, *Phillyrea*, *Pistacia*, *Quercus ilex* type, *Vitex agnus-castus,* [cf. *Ceratonia*]
Montane taxa	*Abies*, *Picea*, *Betula*, *Fagus*, *Cedrus*, *Picea*, [*Picea omorika*, *Tsuga*, cf. *Larix*]
Pioneer taxa	*Hippophaë*, *Juniperus*, *Populus*, *Salix*
Shrubs	*Cistus*, Ericaceae, Cannabaceae, *Elaeagnus*
Steppic taxa	*Artemisia*, Chenopodiaceae, *Ephedra fragilis* type, *E. distachya* type, *Lygeum spartum*
Temperate taxa	*Acer, Alnus, Buxus, Carpinus betulus, Ostrya* type, *Castanea, Celtis, Cornus mas, Corylus, Frangula alnus, Fraxinus excelsior* type, *F. ornus, Hedera, Ilex aquifolium, Juglans, Ligustrum, Lonicera, Loranthus europaeus, Parrotia, Platanus, Pterocarya, Quercus robur/cerris* types, *Rhamnus* type, *Sorbus* group, *Sambucus nigra, S. ebulus, Tamarix, Taxus, Tilia, Ulmus, Viburnum, Viscum, Vitis, Zelkova*, [*Aesculus, Carya,* cf. *Liquidambar*, cf. *Morus*]

Supplementary Table 4. Age-depth tie points of the orbital tuning of the composite record from Tenaghi Philippon spanning the past c. 500 kyrs.

Composite depth (m)	Age (kyrs)
0.00	0.00
0.76	1.95
1.79	4.20
3.41	5.79
5.08	8.27
7.46	22.02
7.61	22.02
9.70	29.09
12.64	39.85
12.89	39.85
17.00	60.00
19.78	72.00
22.00	81.00
24.04	95.50
26.00	101.00
29.00	108.00
32.00	124.00
33.50	130.00
35.00	134.00
39.00	166.00
42.00	180.00
43.50	193.00
46.68	218.00
48.85	226.00
50.05	236.00
51.90	257.00
53.00	269.00
55.00	283.00
56.00	288.00
61.00	311.00
68.20	341.00
68.80	351.00
71.80	369.00
76.00	407.00
80.00	428.00
86.00	468.00

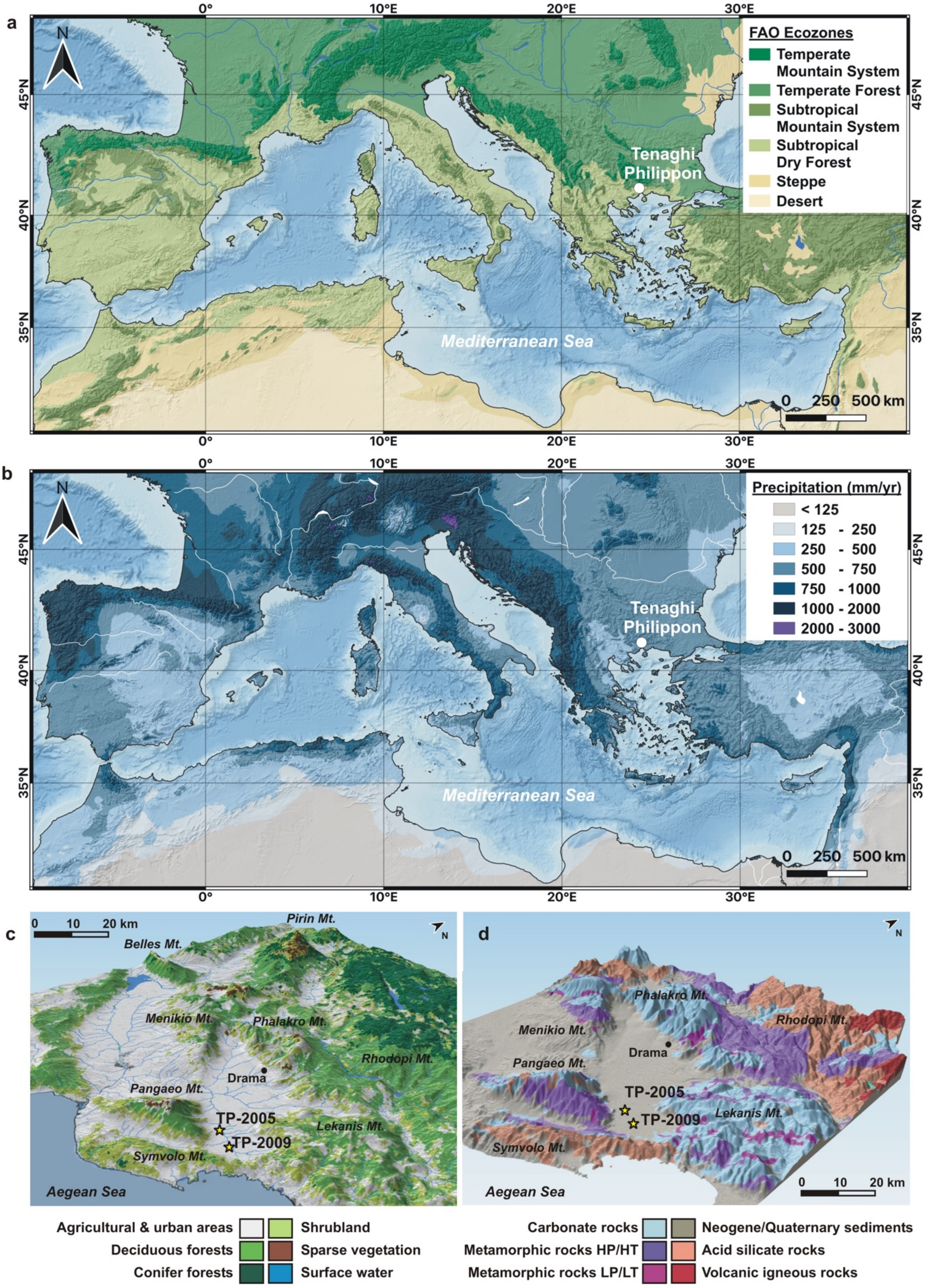

Supplementary Figure 1. Location of Tenaghi Philippon in the Mediterranean region and position of drill sites at Tenaghi Philippon. a, Major vegetation biomes following the Food and

Agriculture Organization of the United Nations (FAO) ecological zone classification (retrieved from https://data.apps.fao.org/map/catalog/srv/eng/catalog.search#/metadata/baa463d0-88fd-11da-a88f-000d939bc5d8) (ref. 7). b, Annual precipitation data for the period 1970–2000 (retrieved from WorldClim version 2.1 climate dataset: https://www.worldclim.org/data/worldclim21.html)(ref. 8). c and d, Topographic and geological maps of the Drama Basin and surrounding mountains indicating the location of the TP-2005 and TP-2009 drill sites; dominant vegetation biomes according to the Ecological Land Units of the United States Geological Survey (retrieved from https://rmgsc.cr.usgs.gov/ecosystems/datadownload.shtml) (ref. 9), and geological data from the Greek Geological Survey (www.eagme.gr). Topographic and bathymetric data from NASA Shuttle Radar Topography Mission (SRTM) datasets (ref. 10). All maps were generated using QGIS software (Version 3.16.10).

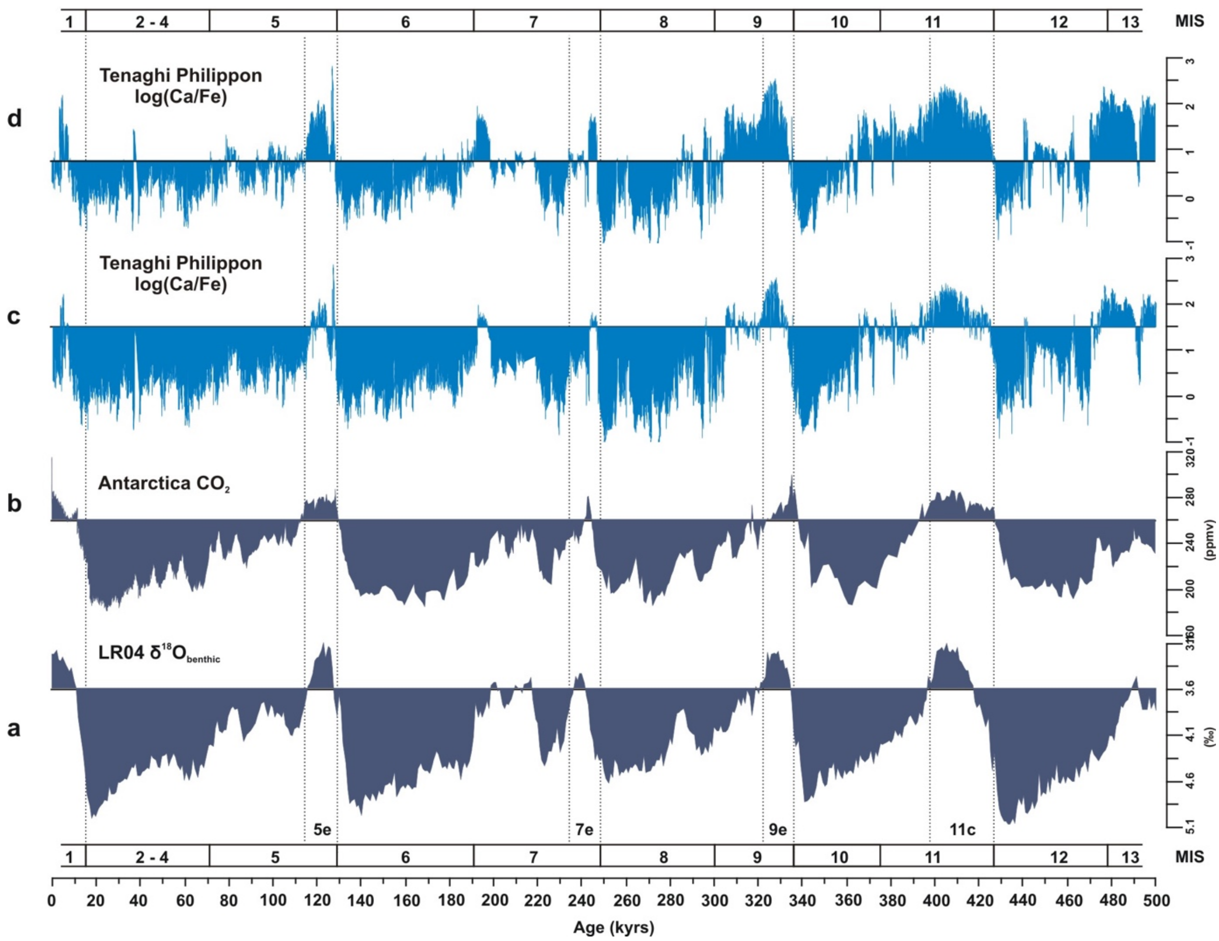

Supplementary Figure 2. XRF-based log(Ca/Fe) values from Tenaghi Philippon and global climate records for the past 500 kyrs. a, LR04 benthic $\delta^{18}O$ stack[11] plotted above and below the 3.6 ‰ threshold for glacial/interglacial conditions[1]. b, Antarctic atmospheric CO_2 (ref. 4) plotted above and below the 260 ppmv threshold for glacial/interglacial conditions[1]. c, and d, log(Ca/Fe) from Tenaghi Philippon plotted above and below the 1.5 and 0.8 values, respectively. Warmest interglacial sub-stages (vertical dashed lines) after ref. (12).

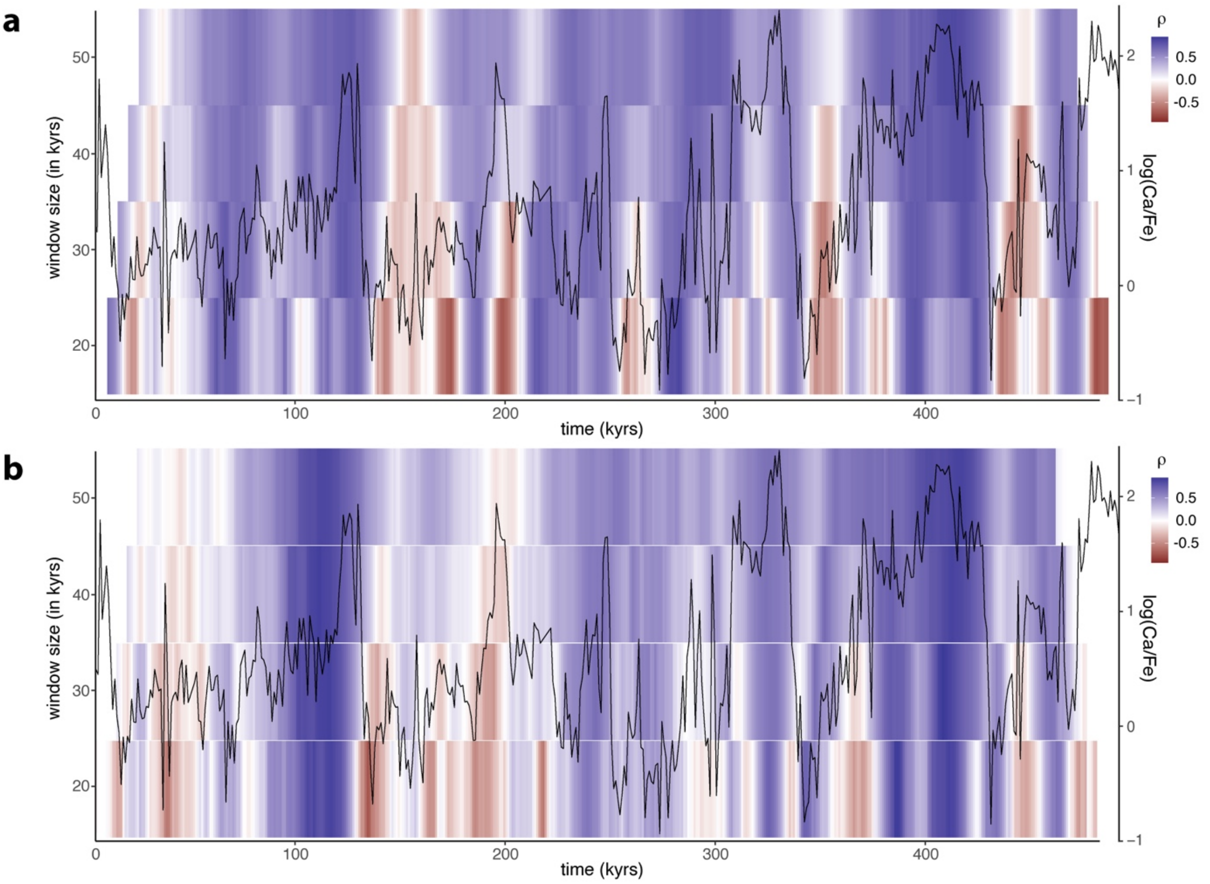

Supplementary Figure 3. ‘Rolling correlations’ as an indicator of changes in the correlation between the Tenaghi Philippon and Antarctic times series through time. a, log(Ca/Fe) against CO_2 (ref. 4). b, log(Ca/Fe) against CH_4 (ref. 3). Applied window sizes of 20 and 50 time steps each representing 1 kyr, increasing in increments of 10. ρ: Spearman‘s correlation coefficient.

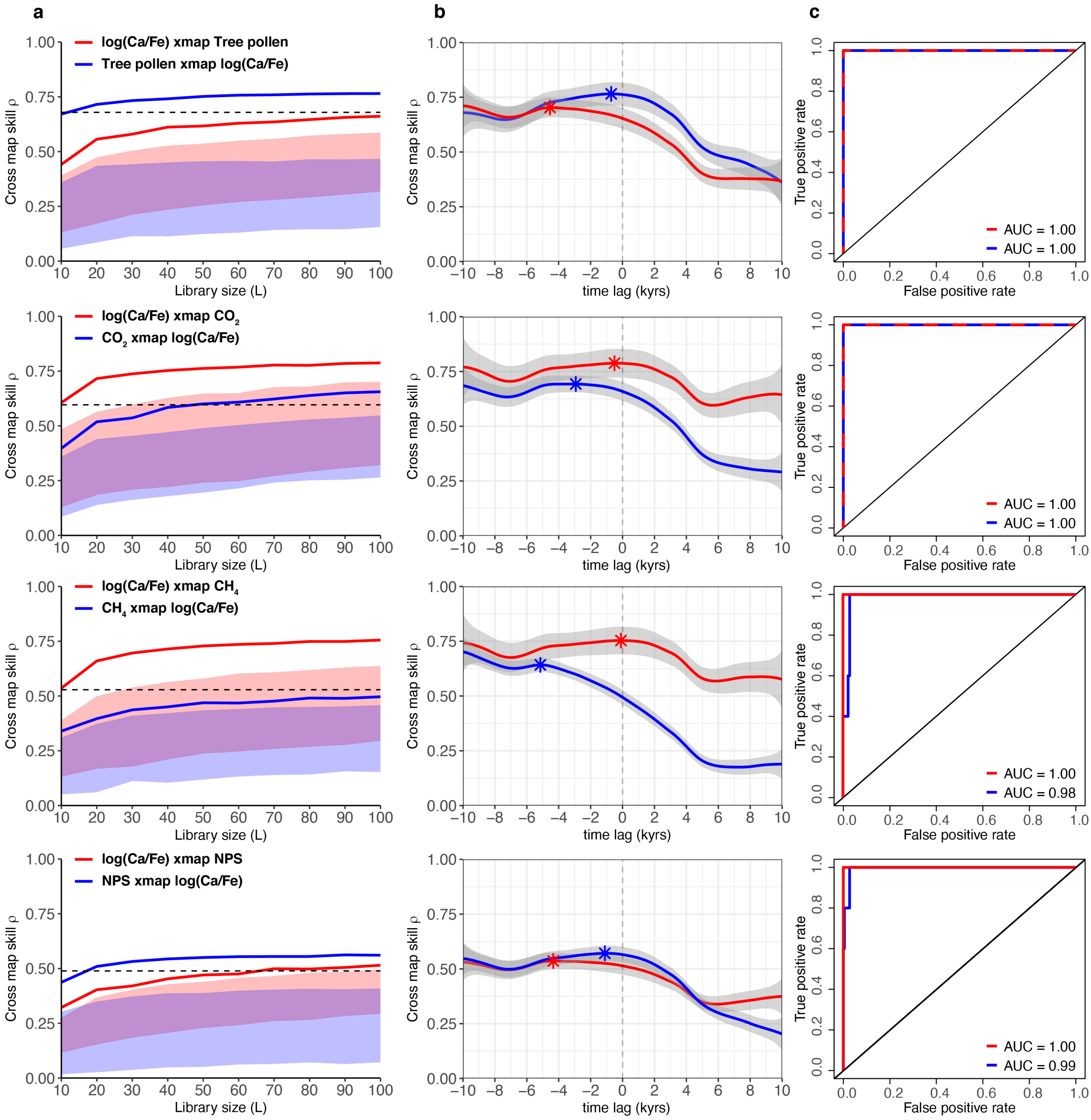

Supplementary Figure 4. Convergent cross-mapping (CCM) results between the Tenaghi Philippon [tree pollen abundances and log(Ca/Fe)] and global proxy timeseries (compare Supplementary Table 2). a, Correlation of cross-mapped against observed values as a function of the timeseries length ('CCM skill'). 'A xmap B' quantifies the causal effect of variable B on variable A by predicting the state of variable A from E lagged time-series fragments of variable B. Shaded areas represent the 5th to 95th percentiles of the 'CCM skill' for 100 surrogate timeseries from the null models. The black dashed line represents the cross-correlation of the two timeseries. b, Time displacements maximizing CCM skill corresponding to the causal relationship among the timeseries. Time displacements are negative when past values of the cross-mapped variable B (*cause*) are estimated by the embedded variable A

(*effect*). Positive time displacements suggest that there is no flow of causal information from variable B (*cause*) to variable A (*effect*), and hence changes in variable B are not reflected in variable A until sometime in the future. Shaded areas represent the 95% confidence interval for predictions from the smoothing model. Color coding as in panel (a). c, Area under curve (AUC) of the sensitivity test results as a measure of performance across all possible threshold settings representing the probability that CCM predictions are ranked correctly (i.e., AUC = 1 represents a 100 % probability of correct predictions). Color coding as in panel (a).

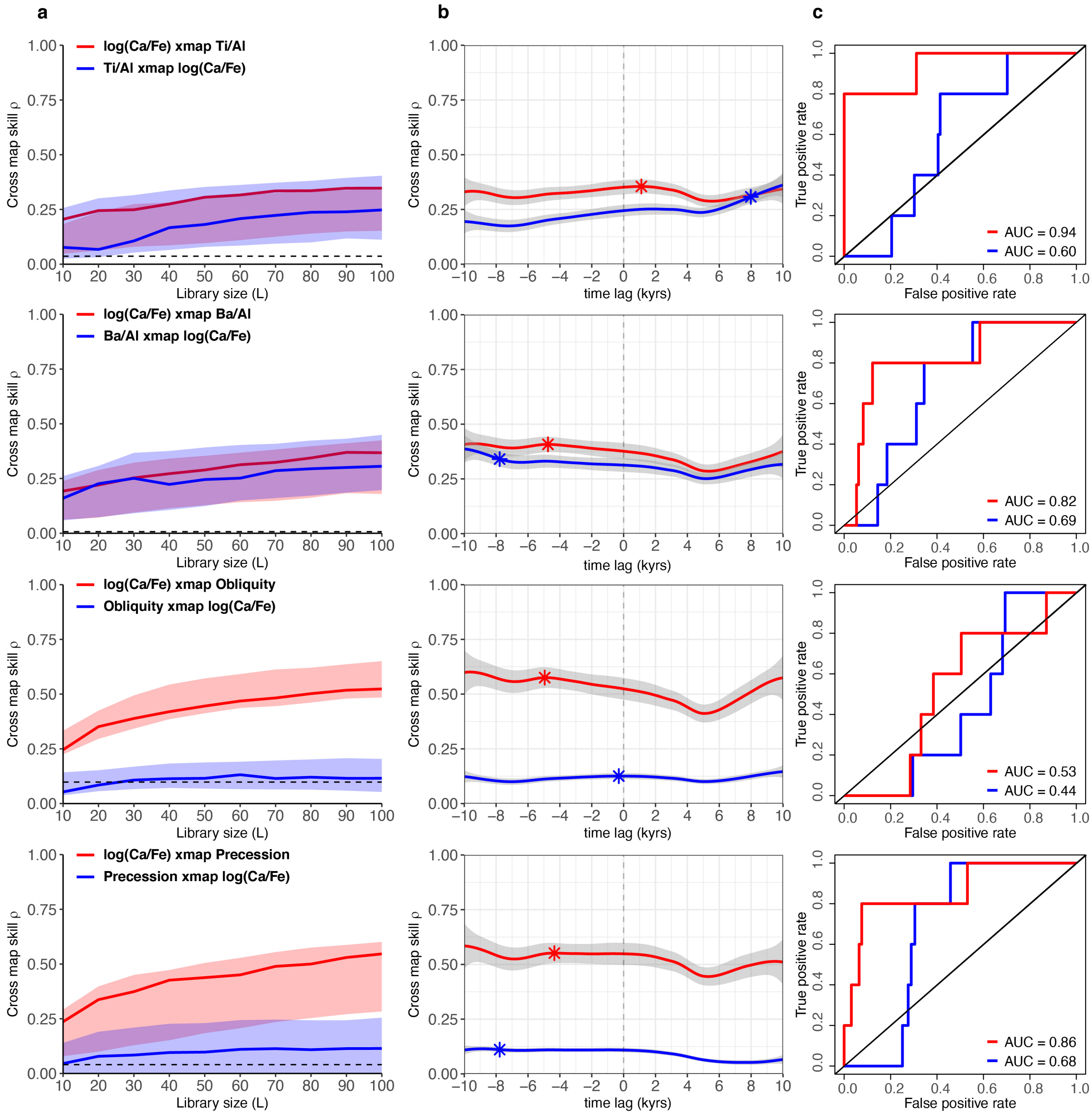

Supplementary Figure 4. *(continued)*

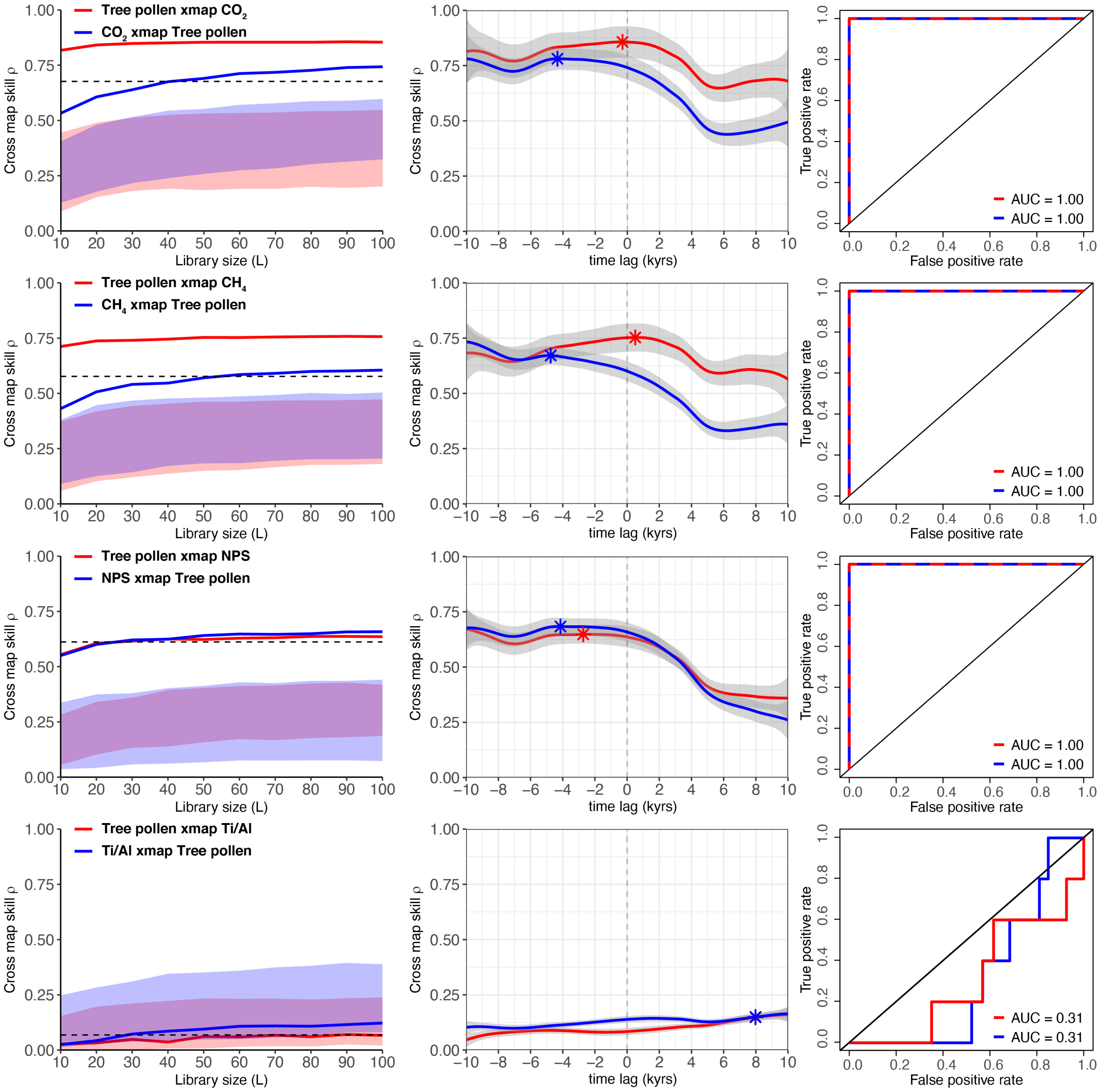

Supplementary Figure 4. *(continued)*

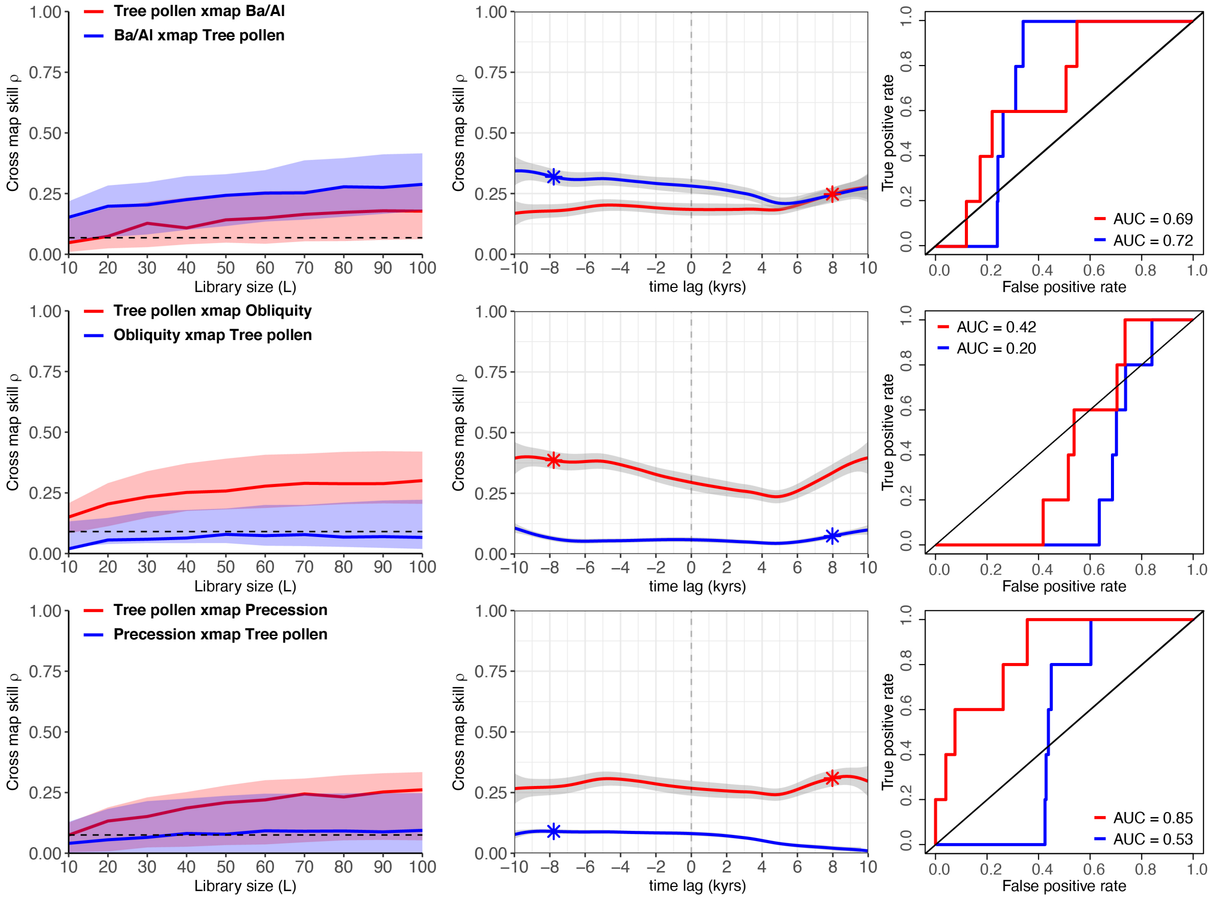

Supplementary Figure 4. *(continued)*

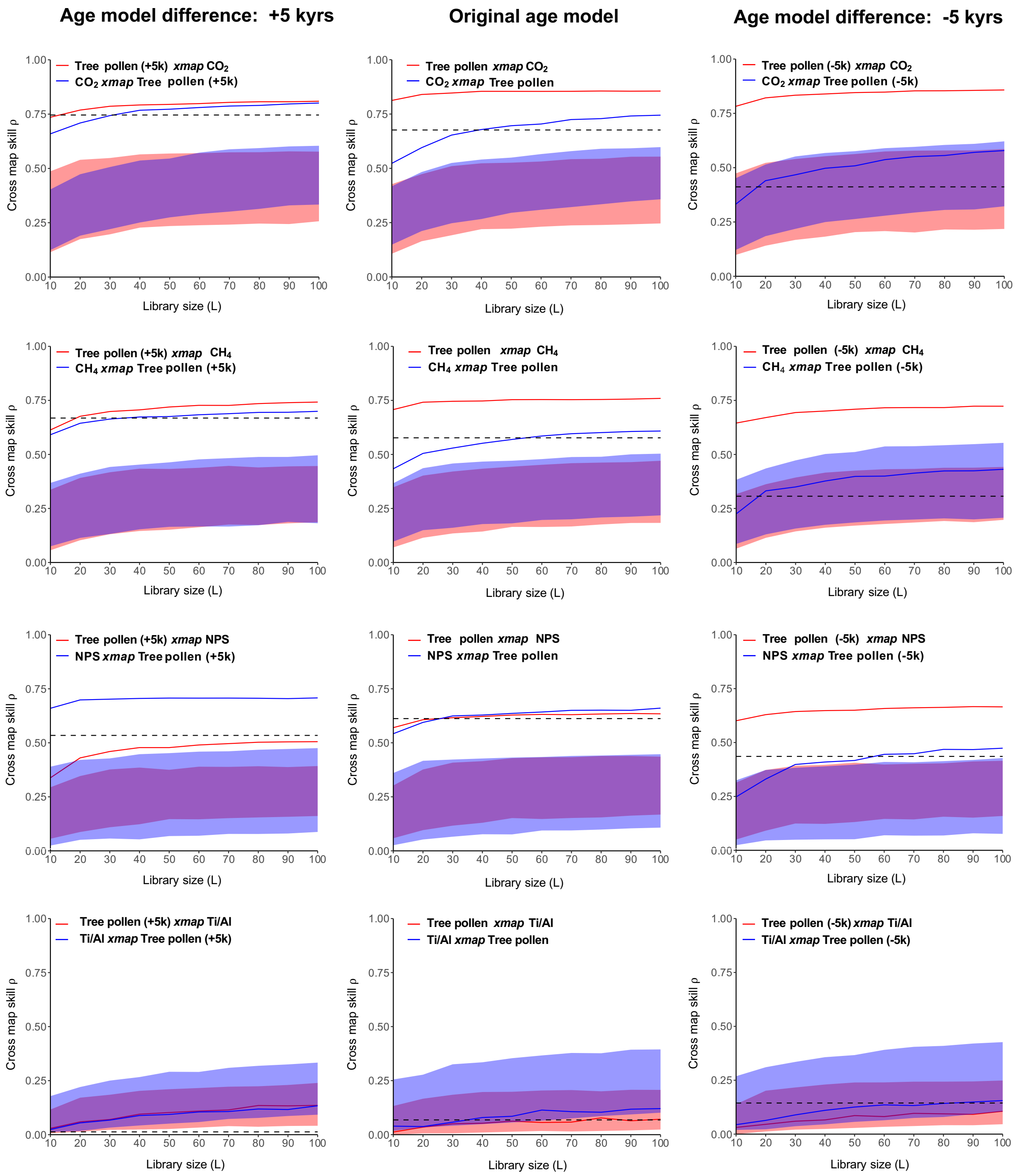

Supplementary Figure 5. Comparison of the convergent cross-mapping (CCM) results between the Tenaghi Philippon [tree pollen abundances and log(Ca/Fe)] and global proxy timeseries using the original age model and two alternative ones shifted by +/- 5 kyrs (i.e., accounting for maximum age uncertainties).

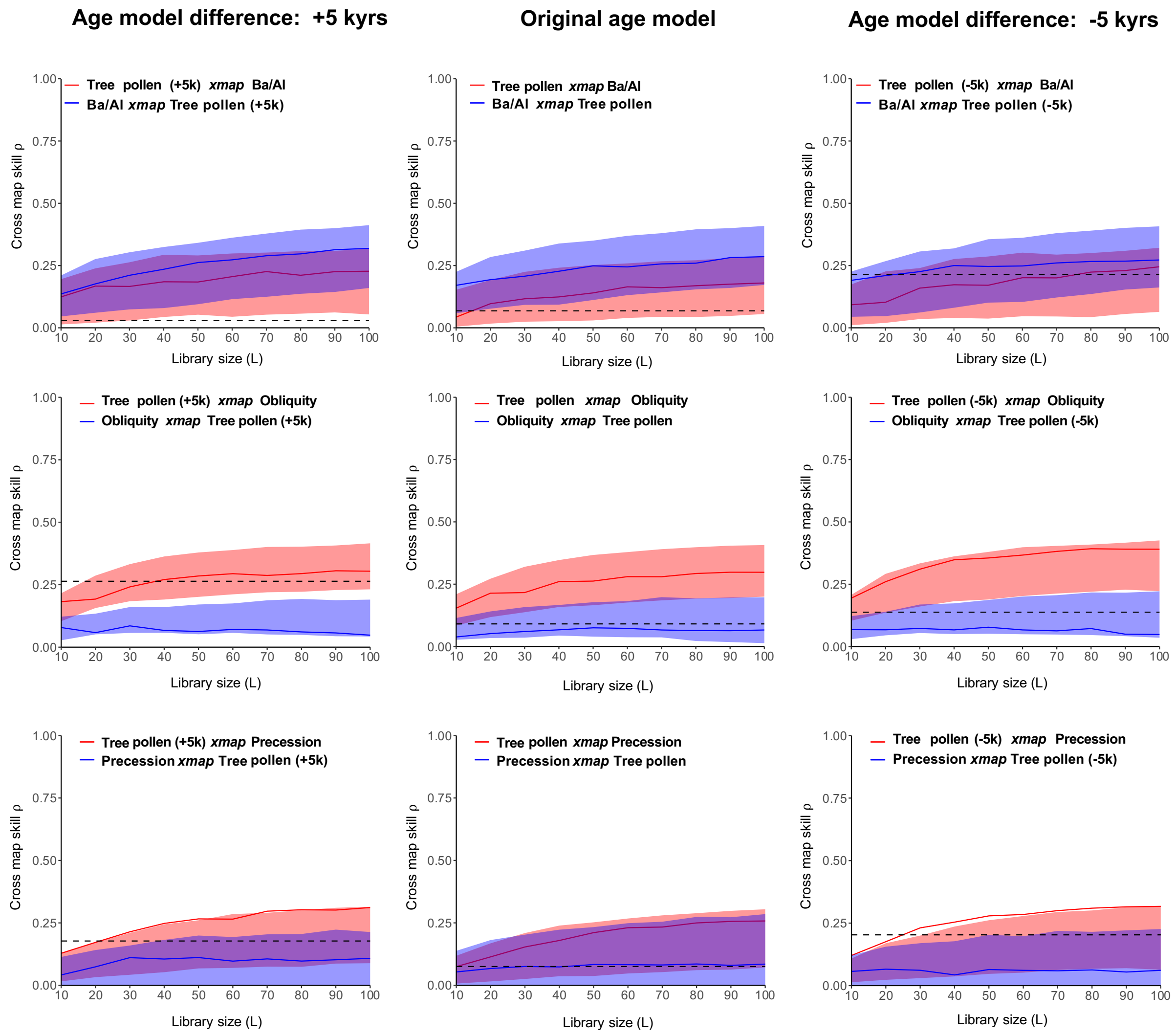

Supplementary Figure 5. *(continued)*

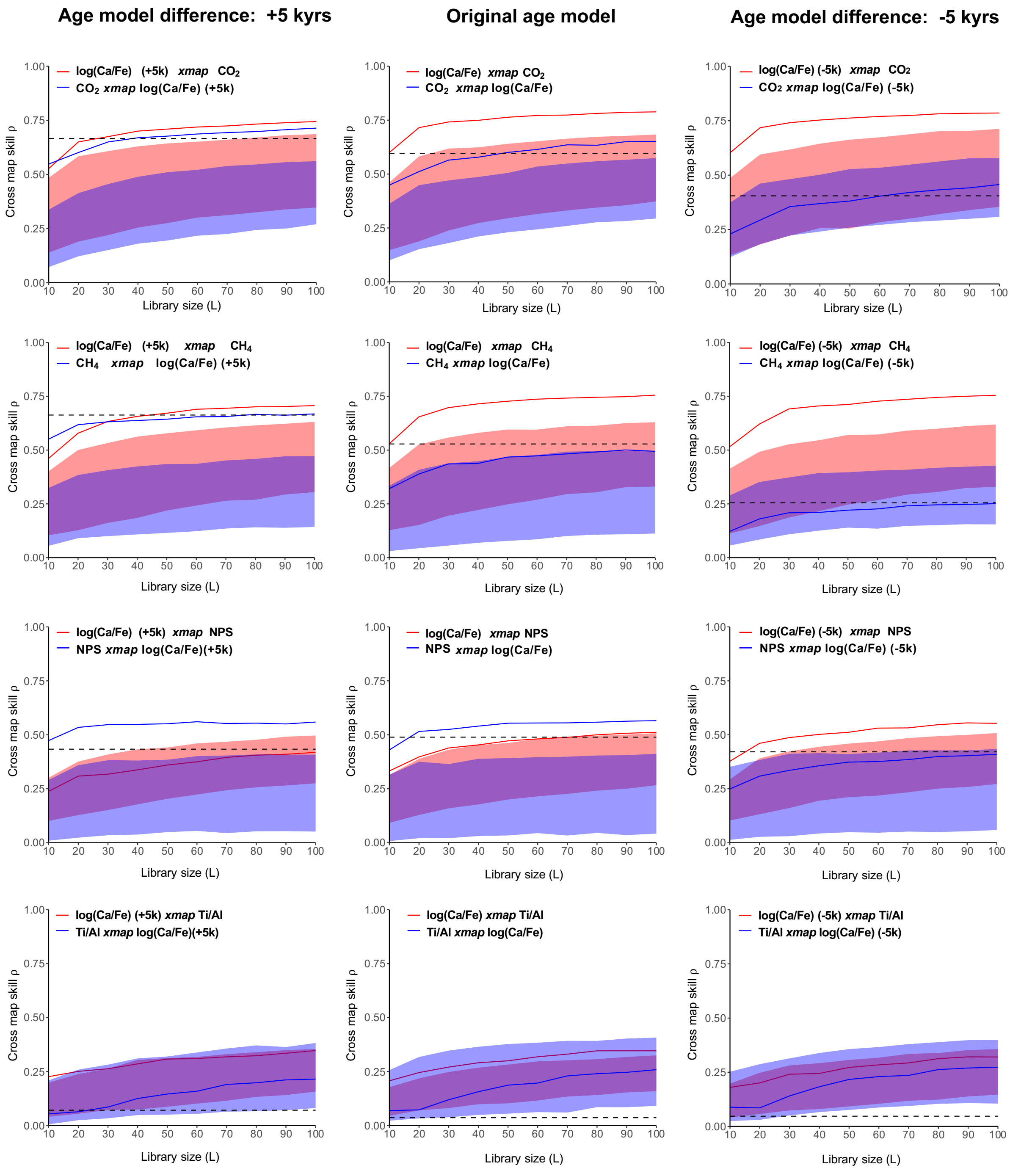

Supplementary Figure 5. *(continued)*

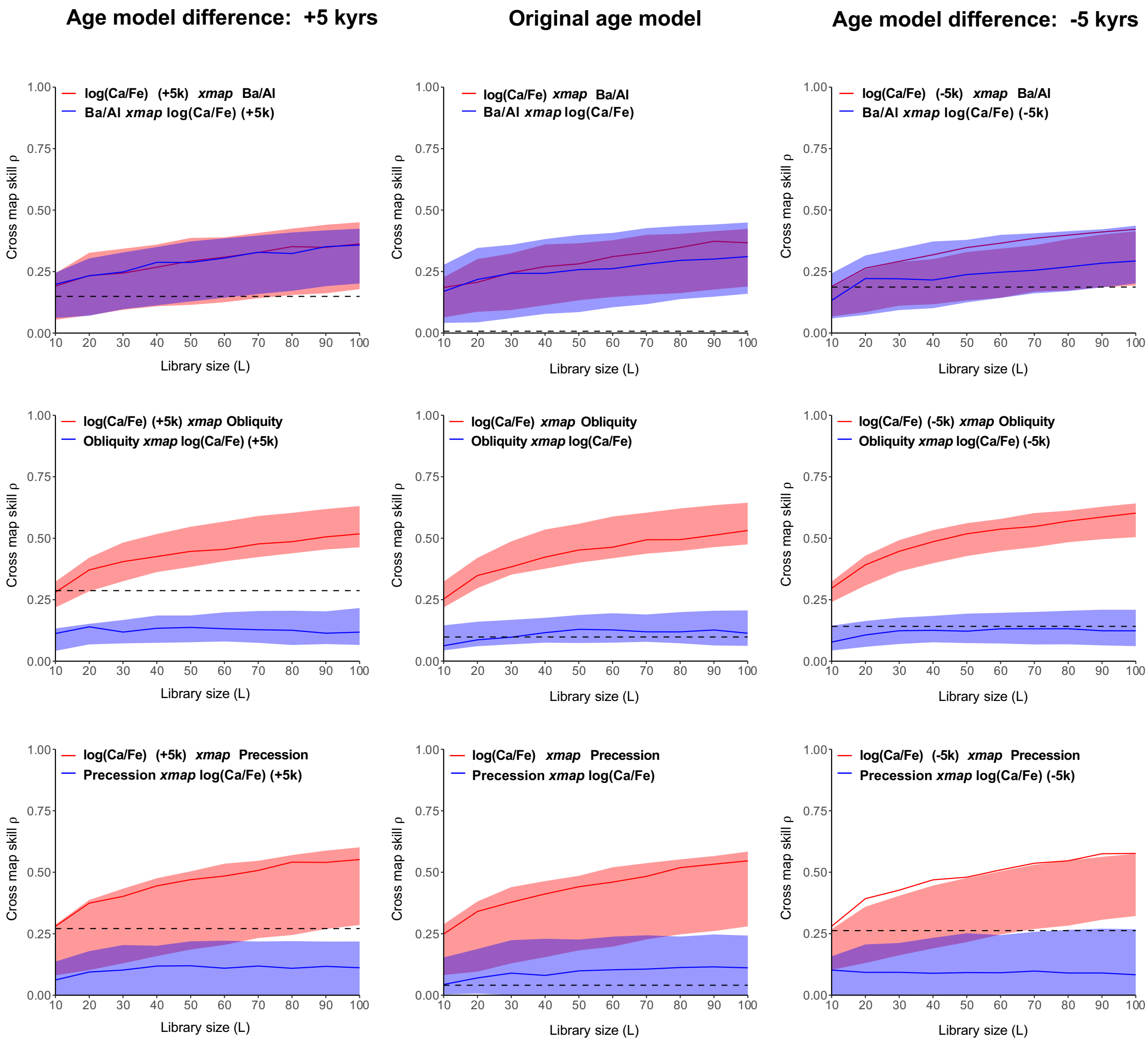

Supplementary Figure 5. *(continued)*

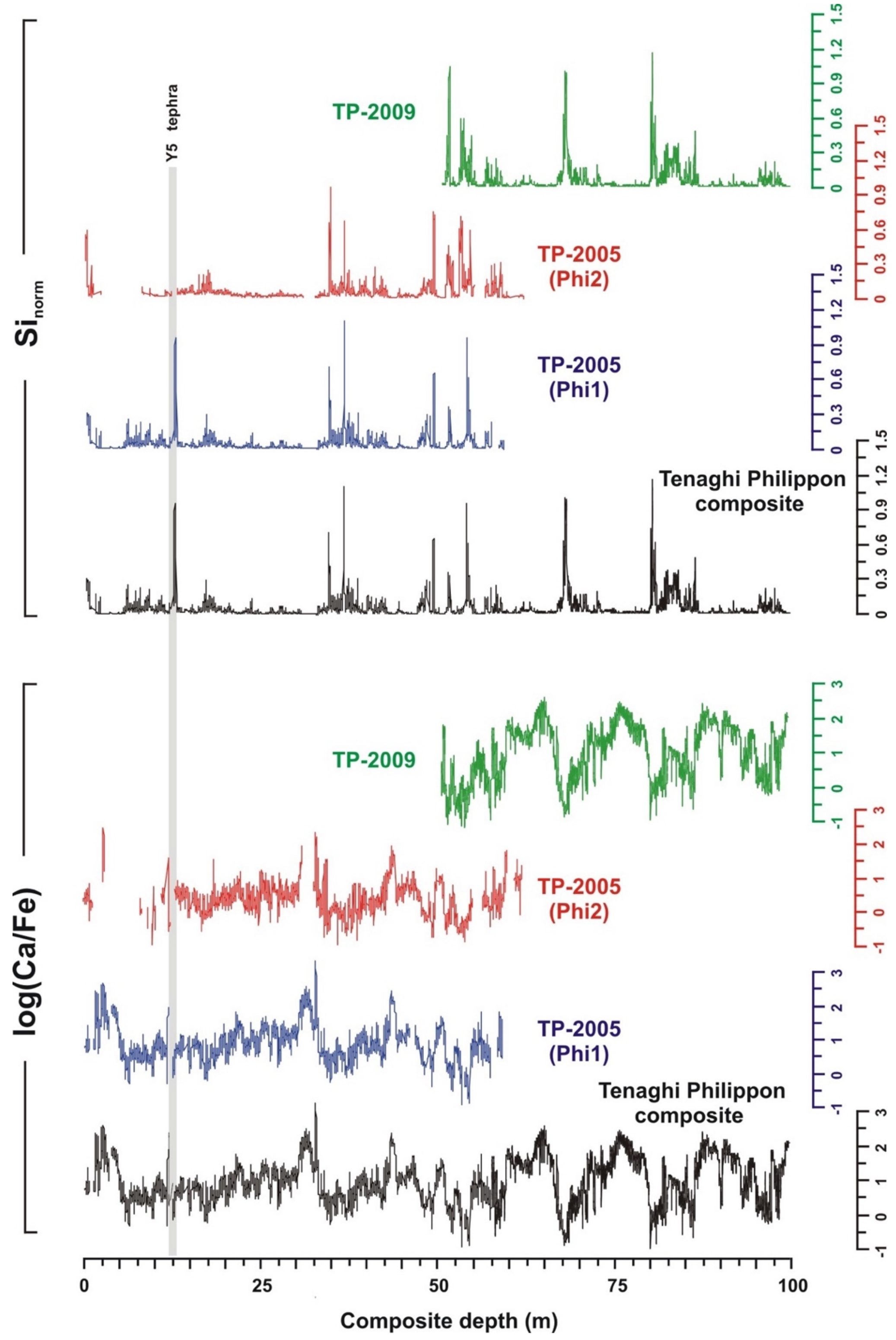

Supplementary Figure 6. Development of the Tenaghi Philippon composite record. XRF-based log(Ca/Fe) and Si records of the TP-2005 (Phi1 and Phi2) and TP-2009 cores plotted against the composite depth scale. The gaps in the TP-2005 (Phi2) core are due to heavy sampling prior to XRF core scanning.

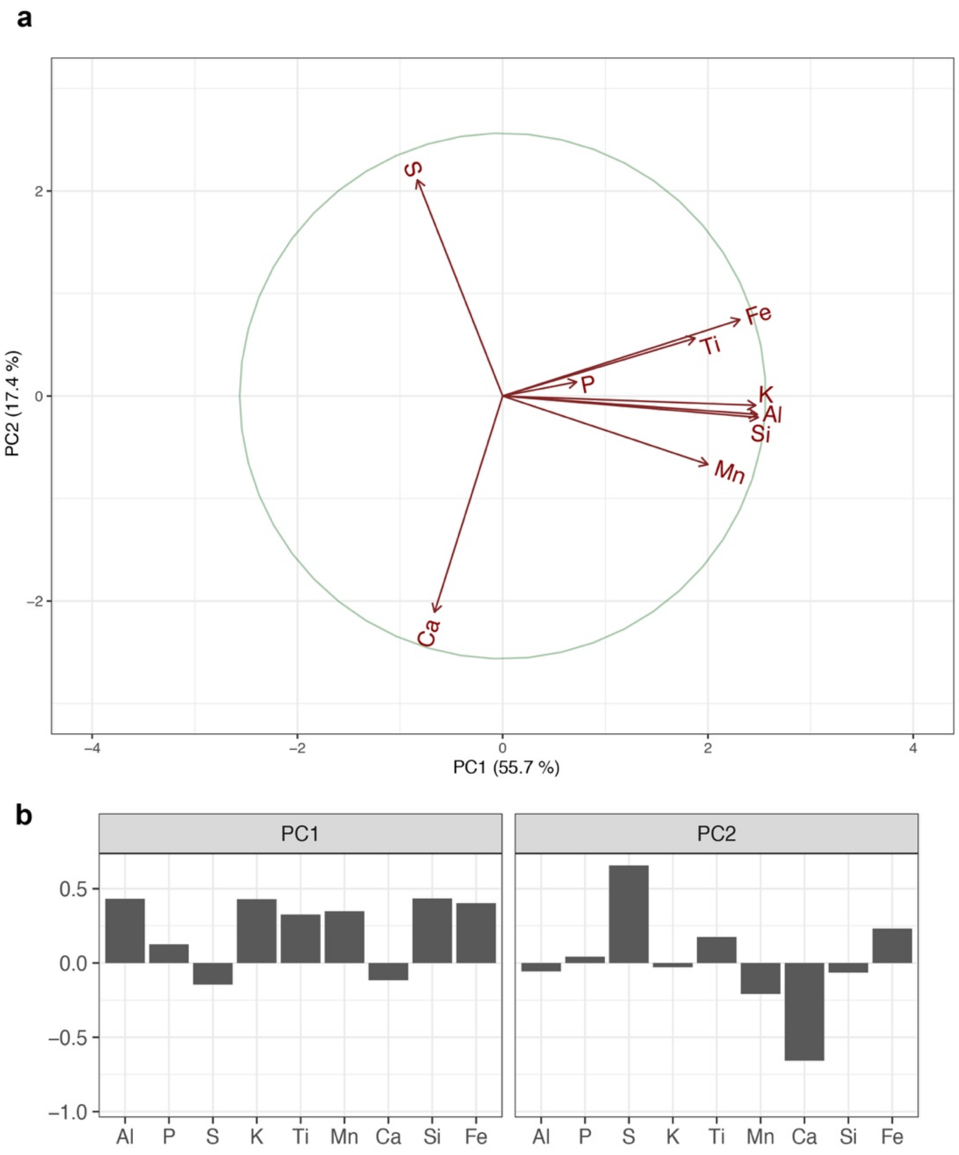

Supplementary Figure 7. Principal component analysis of the XRF-based elemental distribution at Tenaghi Philippon. a, biplot and b, loadings of the first two components (total variance explained: 73.1 %).

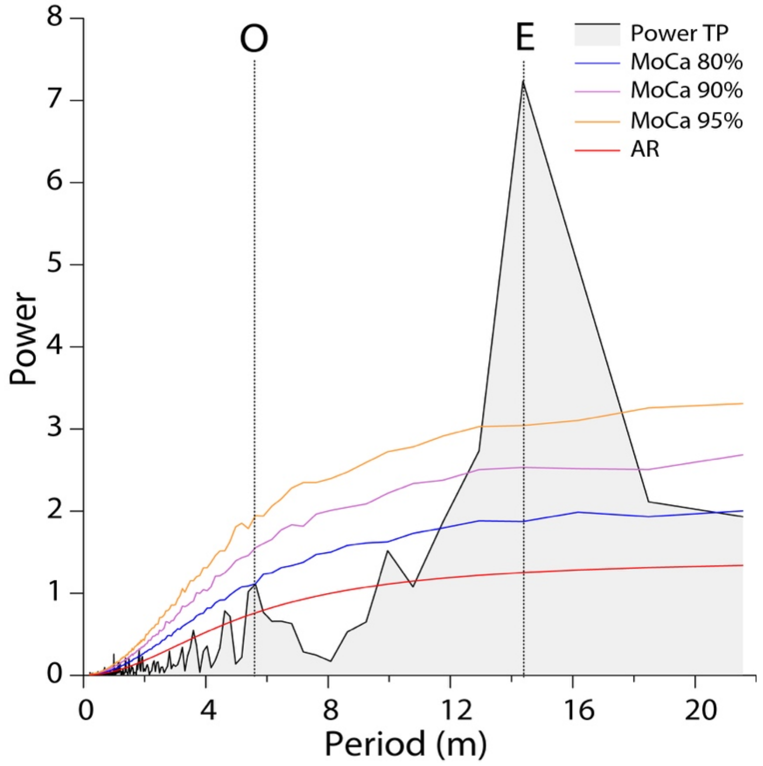

Supplementary Figure 8. Cyclostratigraphy of the XRF-based log(Ca/Fe) record from Tenaghi Philippon. REDFIT analysis[13] was performed on the uppermost 100 m of the composite record testing the significance levels at 80 %, 90 %, and 95 % using a Monte-Carlo simulation. E: Eccentricity; O: Obliquity; MoCa: Monte Carlo; AR: red noise.

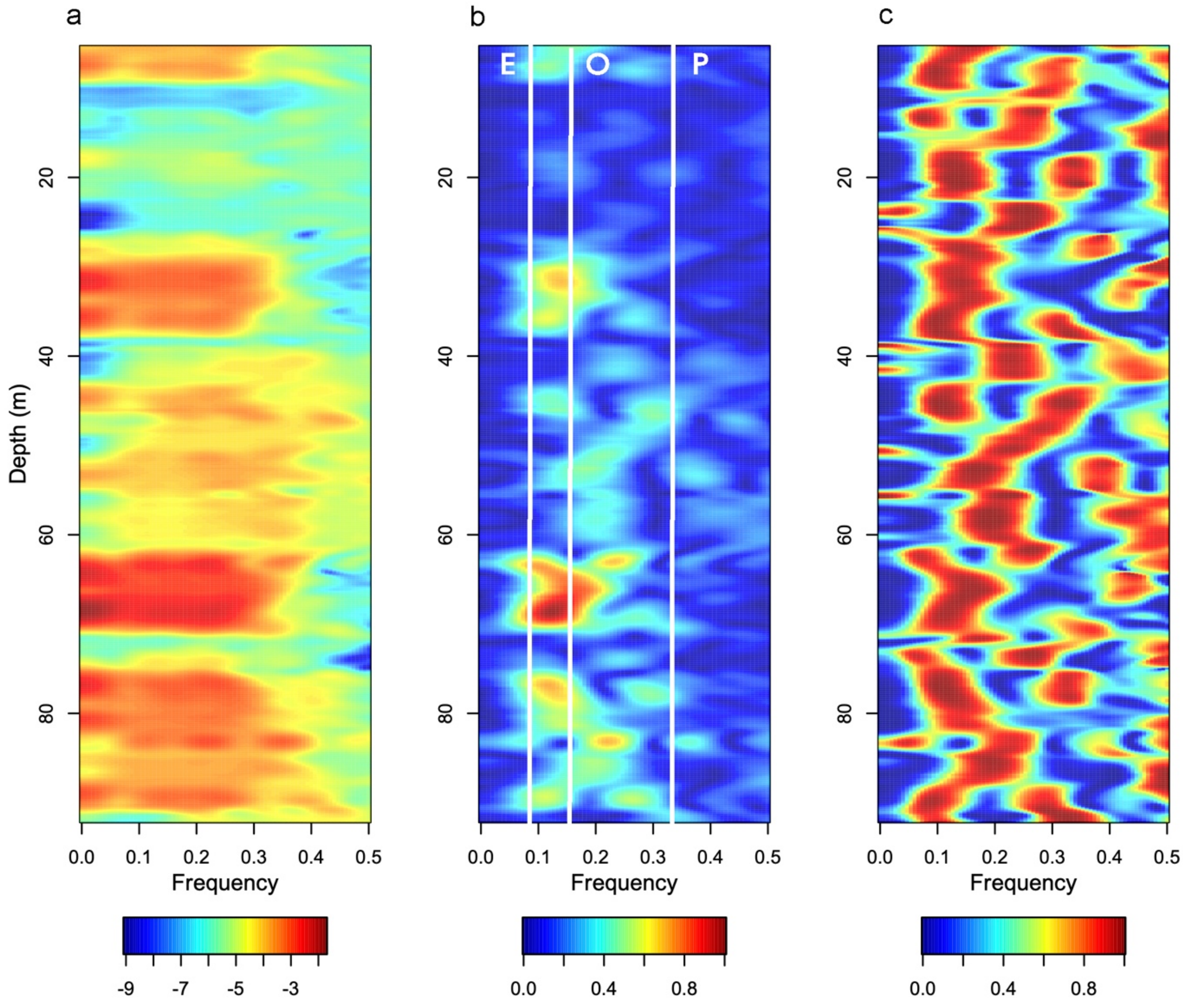

Supplementary Figure 9. Evolutive harmonic spectral analysis (EHA) of the XRF-based log(Ca/Fe) record from Tenaghi Philippon. Frequency test spectrum was performed on the uppermost 100 m of the composite record using the multitaper method[14]. a, Evolutive power spectral analysis (EPSA) in log power. b, Normalized amplitude of EHA. c, Harmonic F-test CL of EHA. E: Eccentricity; O: Obliquity; P: Precession.

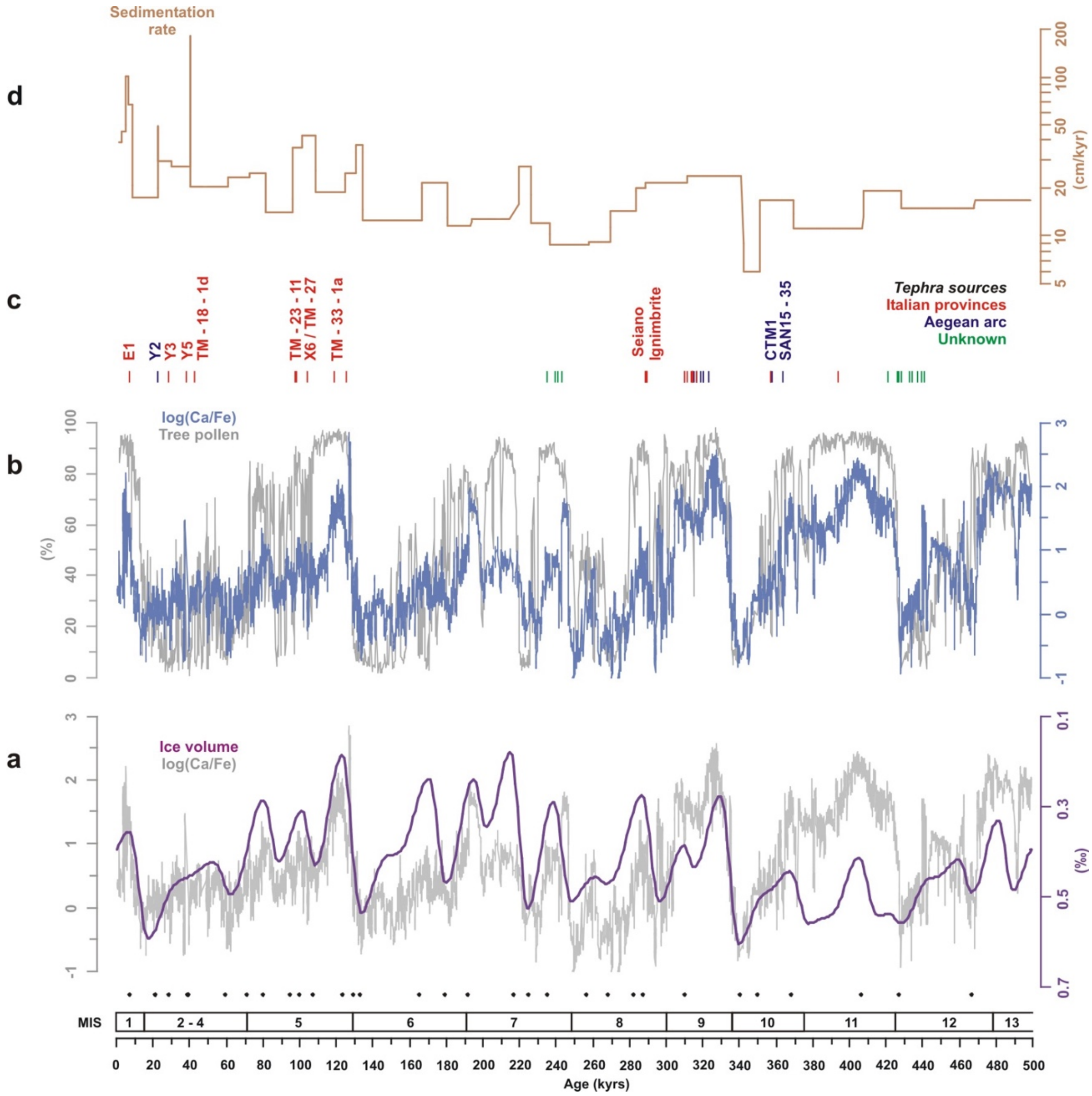

Supplementary Figure 10. Chronology of the Tenaghi Philippon record for the past 500 kyrs. a, ice volume model output from ref. (15). b, log(Ca/Fe) ratio and tree-pollen percentages. c, tephrostratigraphy, with color coding for the different volcanic sources following refs. (16–18). d, Sedimentation rate; the two peaks during MIS 2–4 mark the deposition of the Y2 and Y5 tephras. Tie points marked with black circles. Marine Isotope Stage (MIS) boundaries adapted from ref. (11).

Supplementary References

1. Past Interglacials Working Group of PAGES. Interglacials of the last 800,000 years. *Reviews of Geophysics* **54**, 162-219, doi:10.1002/2015rg000482 (2016).
2. Laskar, J. *et al.* A long-term numerical solution for the insolation quantities of the Earth. *Astronomy and Astrophysics* **428**, 261-285, doi:10.1051/0004-6361:20041335 (2004).
3. Loulergue, L. *et al.* Orbital and millennial-scale features of atmospheric CH_4 over the past 800,000 years. *Nature* **453**, 383-386, doi:10.1038/nature06950 (2008).
4. Bereiter, B. *et al.* Revision of the EPICA Dome C CO_2 record from 800 to 600 kyr before present. *Geophysical Research Letters* **42**, 542-549, doi:10.1002/2014GL061957 (2015).
5. Barker, S. *et al.* Early interglacial legacy of deglacial climate instability. *Paleoceanography and Paleoclimatology* **34**, 1455-1475, doi:10.1029/2019PA003661 (2019).
6. Grant, K. M. *et al.* A 3 million year index for North African humidity/aridity and the implication of potential pan-African Humid periods. *Quaternary Science Reviews* **171**, 100-118, doi:10.1016/j.quascirev.2017.07.005 (2017).
7. Food and Agriculture Organization of the United Nations. *Global Forest Resources Assessment 2000: Main Report*. FAO (2000).
8. Fick, S. E. & Hijmans R. J. WorldClim 2: new 1km spatial resolution climate surfaces for global land areas. *International Journal of Climatology* **37**, 4302-4315, doi.org/10.1002/joc.5086 (2017).
9. Sayer, R. *et al.* A new map of global ecological land units — An ecophysiographic stratification approach. Geosciences and Environmental Change Science Center, Land Change Science. Association of American Geographers, Washington D.C., USA (2014).
10. NASA JPL *NASA Shuttle Radar Topography Mission Global 1 arc second* [Dataset]. NASA EOSDIS Land Processes DAAC. Accessed 2022-12-12 from https://doi.org/10.5067/MEaSUREs/SRTM/SRTMGL1.003 (2013).
11. Lisiecki, L. E. & Raymo, M. E. A Pliocene-Pleistocene stack of 57 globally distributed benthic $\delta^{18}O$ records. *Paleoceanography* **20**, PA1003, doi:10.1029/2004pa001071 (2005).
12. Tzedakis, P. C. *et al.* Can we predict the duration of an interglacial? *Climate of the Past* **8**, 1473-1485, doi:10.5194/cp-8-1473-2012 (2012).
13. Schulz, M. & Mudelsee, M. REDFIT: estimating red-noise spectra directly from unevenly spaced paleoclimatic time series. *Computers & Geosciences* **28**, 421-426 doi:10.1016/S0098-3004(01)00044-9 (2002).
14. Thomson, D. J. Spectrum estimation and harmonic analysis. *Proceedings of the IEEE* **70**, 1055-1096, doi:10.1109/PROC.1982.12433 (1982).

15. Imbrie, J. & Imbrie, J. Z. Modeling the climatic response to orbital variations. *Science* **207**, 943-953, doi:10.1126/science.207.4434.943 (1980).

16. Vakhrameeva, P. *et al.* The cryptotephra record of the Marine Isotope Stage 12 to 10 interval (460–335 ka) at Tenaghi Philippon, Greece: Exploring chronological markers for the Middle Pleistocene of the Mediterranean region. *Quaternary Science Reviews* **200**, 313-333, doi:10.1016/j.quascirev.2018.09.019 (2018).

17. Wulf, S. *et al.* The marine isotope stage 1–5 cryptotephra record of Tenaghi Philippon, Greece: Towards a detailed tephrostratigraphic framework for the Eastern Mediterranean region. *Quaternary Science Reviews* **186**, 236-262, doi:10.1016/j.quascirev.2018.03.011 (2018).

18. Vakhrameeva, P. *et al.* Eastern Mediterranean volcanism during marine isotope stages 9 to 7e (335–235 ka): Insights based on cryptotephra layers at Tenaghi Philippon, Greece. *Journal of Volcanology and Geothermal Research* **380**, 31-47, doi:10.1016/j.jvolgeores.2019.05.016 (2019).
